# Bioactive expression of eukaryotic cytochrome P450 ferulate-5-hydroxylase in *Escherichia coli* for sustainable synthesis of antioxidant 5-hydroxyferulic acid

**DOI:** 10.1186/s40643-025-00919-z

**Published:** 2025-07-15

**Authors:** Ping Sun, Yuan Tian, Luyi Wang, Pengcheng Chen, Dan Wu, Pu Zheng

**Affiliations:** School of Biotechnology and Key Laboratory of Industrial Biotechnology of Ministry of Education, Jiangnan University, Wuxi, 214122 China

**Keywords:** Ferulate-5-Hydroxylase, Biotransformation, 5-Hydroxyferulic acid, Ferulic acid, N-Terminal modification

## Abstract

The eukaryotic cytochrome P450 ferulate-5-hydroxylase (F5H), a membrane-bound protein, plays a critical role in lignin synthesis involved in the biosynthesis of 5-Hydroxyferulic acid (5-HFA) from ferulic acid, with 5-HFA offering enhanced antioxidant properties. However, there is challenging to engineer recombinants for 5-HFA synthesis by expressing F5H in *Escherichia coli*. In this work, we co-expressed F5H derived from *Arabidopsis thaliana* (*At*F5H) and NADPH-dependent cytochrome P450 reductase (CPR) in *E. coli*, and successfully synthesized *ortho*-hydroxylated ferulic acid. Simultaneously, by modifying the N-terminal regions of membrane proteins, we increased the concentration of product 5-HFA to 63.6 mg/L. Ferulic acid, utilized as the substrate, was extracted from discarded agricultural by-products, demonstrating a sustainable approach to valorizing agricultural waste. This work also advances the application of plant-derived membrane-bound proteins for the production of plant secondary metabolites in *E. coli*.

**Graphical Abstract:**

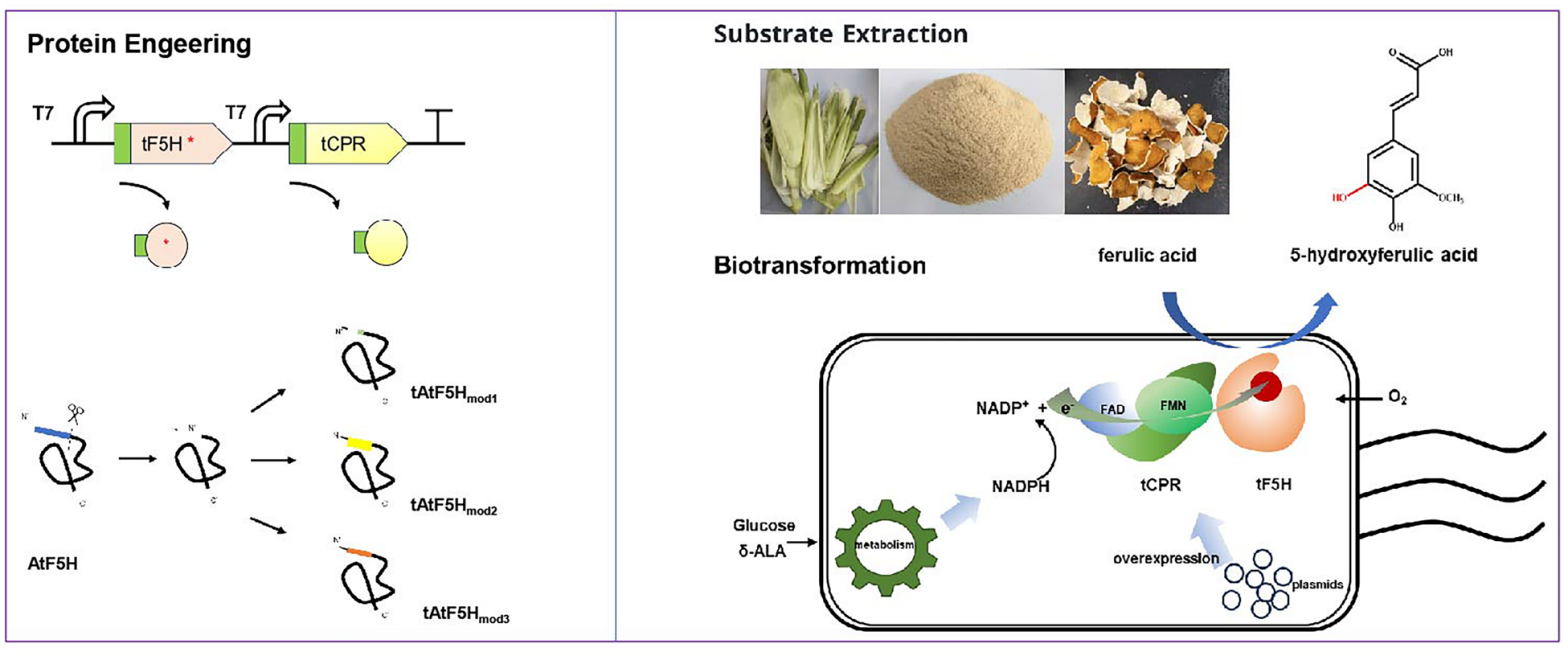

**Supplementary Information:**

The online version contains supplementary material available at 10.1186/s40643-025-00919-z.

## Introduction

In the realm of sustainable biotechnology, the efficient utilization of agricultural byproducts stands as a pivotal area of research. These byproducts, including straw, husks, and other biomass wastes, are not only abundant in resources but are still underutilized, when transformed, can significantly mitigate environmental impacts while providing economic benefits. Among various biomass biomolecules, ferulic acid (FA) is broadly present and is particularly important due to its bioactivities and the prospect of a wide range of application in the pharmaceutical and food industries.

5-Hydroxyferulic acid (5-HFA), an *ortho*-hydroxylated derivative of FA, exhibits stronger antioxidant capacity and water solubility than its precursor (Kylli et al. 2008; Amić et al. 2021). Despite its presence in fruits and cereals, 5-HFA is available only in small quantities, which limits its practical applications (Suzuki et al. 1997; Luo et al. 2011; Lopatriello et al. 2017; Topčagić et al. 2022). Currently, 5-HFA has been reported to be produced from ferulic acid only by 4-hydroxyphenylacetate 3-hydroxylase (Furuya and Kino 2014) or Ferulate-5-hydroxylase(F5H, EC 1.14.14. B13) (Osakabe et al. 1999; Humphreys et al. 1999). In plants, F5H is a cytochrome P450-dependent monooxygenase responsible for converting FA into 5-HFA. It was first detected in xylem extract of lignified poplar that could add hydroxyl group to C5 of the phenyl ring of FA (Grand 1984). F5H is essential for regulating the S/G lignin ratio, crucial for enhancing biomass utilization in areas like ruminant digestibility, chemical pulping, and biofuel production (Weng et al. 2008; Li et al. 2008; Cao et al. 2022; Huntley et al. 2003; Portilla Llerena et al. 2024; Meyer et al. 1998).

Despite its potential, the application of F5H in biotechnological processes faces challenges, primarily due to the intrinsic complexity of eukaryotic P450s, which are difficult to functionally express in bacterial systems (Torres Pazmiño et al. 2010). This complexity has limited the practical utilization of P450-dependent monooxygenases like F5H. F5H, a heme-thiolate oxygenase in the CYP84 subfamily (Meyer et al. 1996) found in the endoplasmic reticulum of plants such as *Arabidopsis* (Chapple et al. 1992), tobacco, and poplar (Franke et al. 2000), catalyzes the hydroxylation of substrates, a process that requires electrons provided by NADPH-cytochrome P450 reductase (CPR). In 1999, Osakabe (Osakabe et al. 1999) successfully heterologously expressed F5H from *Liquidambar formosana* (CAld5H) in engineered *Saccharomyces cerevisiae* INVSc2, enabling the conversion of ferulic acid to 5-HFA. In the same year, Humphreys (Humphreys et al. 1999) expressed bioactive F5H from *Arabidopsis thaliana* (*At*F5H) in engineered *Saccharomyces cerevisiae* WAT11, marking a significant advancement in the biosynthesis of 5-HFA. However, the expression of bioactive F5H in *E. coli* for 5-HFA synthesis remains unachieved. Recent studies have focused on optimizing conditions to enhance *At*F5H activity (Jiang and Morgan 2004), employing various electron transfer pathways crucial for efficient catalysis (Gou et al. 2019; Zhao et al. 2023).

In this study, we constructed recombinants by co-expressing truncated F5H and the truncated CPRs in *E. coli* for 5-HFA synthesis. Then, N-terminal modifications of CPR and F5H were implemented to enhance the production of 5-HFA. Additionally, this study employed an innovative approach by extracting FA from inexpensive and abundant agricultural by-products, serving as substrates for 5-HFA synthesis, thereby achieving efficient resource utilization. Finally, by optimizing fermentation and transformation conditions, we aimed to maximize the yield of 5-HFA, thus improving the overall efficiency of the production process.

## Materials and methods

### Chemicals and reagents

Standard ferulic acid was purchased from Xi’an Guanyu Biotech Co. Ltd. Standard 5-Hydroxyferulic acid was purchased from PhytoLab and isopropyl-*β*-Dthiogalactopyranoside (IPTG) was obtained from Sangon Biotech (Shanghai). HPLC-grade methanol was purchased from Adamas-beta. All other chemicals used were of analytical grade unless specified noted.

### Strain and plasmid construction

In this study, *E. coli* DH5α was served as the cloning strain for routine gene-cloning works and *E. coli* BL21(DE3) was used as the protein expression host for 5-HFA-producing strain construction. The genes encoding F5Hs and CPRs were codon-optimized and synthesized by GENEWIZ (Suzhou, China). All engineered strains used in this study are listed in Table 1 with brief descriptions, including the constructed plasmids and the restriction sites of the plasmids into which genes were cloned. Oligonucleotide synthesis and DNA sequencing analysis were done by GENEWIZ (Suzhou, China). Restriction enzymes, PrimeSTAR^®^ Max DNA polymerase, T4 DNA ligase and restriction endonucleases were purchased from Takara Bio, Inc. (Dalian, China).

**Table 1:** List of key engineered strains used in this study

Strain	Plasmid	Descriptions	Source
tCtA	pETDuet-tAtF5H-tAtR1	pETDuet-1 carrying gene encoding *tAtF5H* (*Nco* I/*Hind* III) and *tAtR1* (*Nde* I/*Kpn* I)	This study
tC	pETDuet-tAtF5H	pETDuet-1 carrying gene encoding *tAtF5H* (*Nco* I/*Hind* III)	This study
tCA	pETDuet-tAtF5H-tAtR1_mod_	double T7 promoters, f1 ori, Amp^R^, pETDuet-1 carrying gene encoding *tAtF5H* (*Nco* I/*Hind* III) and *tAtR1*_*mod*_ (*Nde* I/*Kpn* I)	This study
AFA	pETDuet-CipA_tAtF5H-CipA_tAtR1_mod_	pETDuet-1 carrying gene encoding *CipAtAtF5H* (*Nco* I/*Hind* III) and *CipAtAtR1*_*mod*_ (*Nde* I/*Kpn* I)	This study
BFA	pETDuet-CipB_tAtF5H-CipB_tAtR1_mod_	pETDuet-1 carrying gene encoding *CipBtAtF5H* (*Nco* I/*Hind* III) and *CipBtAtR1*_*mod*_ (*Nde* I/*Kpn* I)	This study
1CA	pETDuet-tAtF5H_mod1_-tAtR1_mod_	double T7 promoters, f1 ori, Amp^R^, pETDuet-1 carrying gene encoding *tAtF5H*_*mod1*_ (*Nco* I/*Hind* III) and *tAtR1*_*mod*_ (*Nde* I/*Kpn* I)	This study
2CA	pETDuet-tAtF5H_mod2_-tAtR1_mod_	pETDuet-1 carrying gene encoding *tAtF5H*_*mod2*_ (*Nco* I/*Hind* III) and *tAtR1*_*mod*_ (*Nde* I/*Kpn* I)	This study
3CA	pETDuet-tAtF5H_mod3_-tAtR1_mod_	pETDuet-1 carrying gene encoding *tAtF5H*_*mod3*_ (*Nco* I/*Hind* III) and *tAtR1*_*mod*_ (*Nde* I/*Kpn* I)	This study

The targeted fragments amplified via PCR were integrated into the corresponding plasmids using conventional restriction enzyme digestion and ligation methods, and the recombinant plasmids were validated by GENEWIZ (Suzhou, China) through Sanger sequencing (Supplementary Table S1). The correct plasmids were subsequently transformed into the targeted host strains, producing a series of engineered strains. To enable Western blot detection, a 6 × His tag was fused to the C-terminus of AtF5H_mod_s in strains tCA, 1CA, 2CA, and 3CA by GENEWIZ (Fig.S2).

### Medium and shake-flask cultivation conditions and biotransformation of ferulic acid

Luria–Bertani (LB) and modified M9 media were used for *E. coli* cultivation and shake-flask fermentation, respectively. LB medium contained 5 g/L yeast extract, 10 g/L tryptone, and 10 g/L NaCl, whereas the modified M9 medium contained 6.78 g/L Na_2_HPO_4_, 3.0 g/L KH_2_PO_4_, 0.5 g/L NaCl, 1.0 g/L NH_4_Cl, 0.241 g/L MgSO_4_, 0.011 g/L CaCl_2_, and 10 g/L glucose.

Shake-flask fermentation was performed as follows. First, *E. coli* BL21 (DE3) harboring recombinant plasmids were inoculated in LB medium (containing 50 µg/mL ampicillin) and cultured at 37 °C and 220 rpm for approximately 12 h to generate the seed culture. Subsequently, 2% pre-inoculum was inoculated into the shake flask containing 50 mL modified M9 medium with 50 µg/mL ampicillin at 37 °C while being shaken at 220 rpm. When OD_600_ reached approximately 0.6, 0.2 mM isopropyl-*β*-D-thiogalactoside (IPTG) and 0.1 mM δ-Aminolevulinic acid (δ-ALA) was added and further incubated at 20 °C and 220 rpm for 12 h. Finally, 0.1 g/L ferulic acid (added as sodium ferulate, with a ferulic acid to NaOH ratio of 5:1 (w/w)) was supplemented to biosynthesize 5-HFA. The culture was incubated for an additional 12 h at 30 °C, collected, extracted and analyzed by HPLC.

For verification of protein expression, the cultures were harvested before ferulate addition and resuspended in 100 mM potassium phosphate buffer (pH 7.0). The cells were lysed by sonication, and each sample was separated into soluble and insoluble fractions by centrifugation at 10,000 g at 4 °C for 30 min for SDS-PAGE analysis.

### Product identification and quantification

The substrate and hydroxylated products were confirmed by HPLC and LC–MS/MS analysis. ferulic acid and 5-HFA in the reaction solution were quantified by reversed-phase chromatography Amethyst C18‑H (4.6 × 250 mm, 5 μm) on an HPLC instrument (Waters, USA). The reaction products were analyzed by HPLC and quantified by comparing their concentrations with those of standard compounds. Samples (10 μL) were solubilized in the mobile phases A and B (40:60, v/v) and injected into a C18 column (Amethyst C18‑H (4.6 × 250 mm, 5 μm). Mobile phase A was methanol containing 1% glacial acetic acid; mobile phase B was water. The reaction products and substrate were eluted at a flow rate of 0.8 mL/min. The eluates were detected by UV at 322 nm. Compounds were identified by comparing their retention times with those of standard sample. The data were shown as mean ± SD of three biological replicates.

The separated supernatant was analysed by LC–MS/MS (UltiMate 3000 HPLC system coupled to a MALDI-TOF/TOF mass spectrometer (Bruker, Germany)) using the selected ion mode: 5-HFA (m/z = 219). Compounds were identified by comparison with pure compounds with regard to their retention time and the fragmentation spectrum in LC–MS/MS.

### Preparation of crude ferulic acid from agricultural by-products

Agricultural by-products such as wheat bran, corn bran, and pomelo peel are chosen as raw materials for extracting ferulic acid. As shown in Fig. 9a, place them in a 65 °C constant temperature oven to dry to a constant weight. Grind into powder using a grinder and sieve through a 60-mesh screen. Accurately weigh 2 g of powder into a 250 mL Erlenmeyer flask. Add alkaline solution A (containing 15% ammonia, 30% ethanol and 55% 2 M NaOH) at a ratio of 1:20 (w/v). After 36 h at 37 °C and 110 rpm, centrifuge at 10,000 × g for 5 min at room temperature. Collect the supernatant and adjust the pH to below 4 with 6 N HCl. Extract the liquid with ethyl acetate at a 1:1 (v:v) ratio. Collect the organic phase when the liquid mixture is layering. Add an equal volume of ethyl acetate to the remaining liquid and extract once more. Combine the extracts, and perform a reverse extraction by adding 10% volume of saturated NaCl solution. Separate the upper organic mixture and remove water with anhydrous sodium sulfate. Finally, use a rotary evaporator at 40 °C to obtain solid particles. Wash and dissolve the solid particles with an appropriate amount of anhydrous ethanol, centrifuge at 10,000 × g for 5 min at room temperature, and remove the sediment. Use a rotary evaporator at 40 °C again to obtain the solid, which is the crude ferulic acid.

### Optimization of fermentation conditions on 5-HFA Synthesis

To optimize the production of 5-HFA using the engineered strain 2CA, we systematically adjusted several fermentation parameters. IPTG concentrations were tested over a range from 0.1 mM to 0.5 mM, and δ-ALA concentrations from 0.1 mM to 1.2 mM. Induction temperatures were varied between 15 °C and 40 °C to enhance enzyme stability and activity. Additionally, the timing for adding ferulic acid was evaluated between 8 to 24 h post-induction. We also explored biotransformation temperatures from 15 °C to 40 °C to identify the optimal conditions for conversion. Under these optimal settings, the effect of varying ferulic acid concentrations from 0.1 g/L to 1 g/L on 5-HFA production was investigated. These modifications were targeted at maximizing the yield of 5-HFA.

## Results and discussion

### Construction of recombinants by co-expressing F5H and CPR

**Fig.1:**
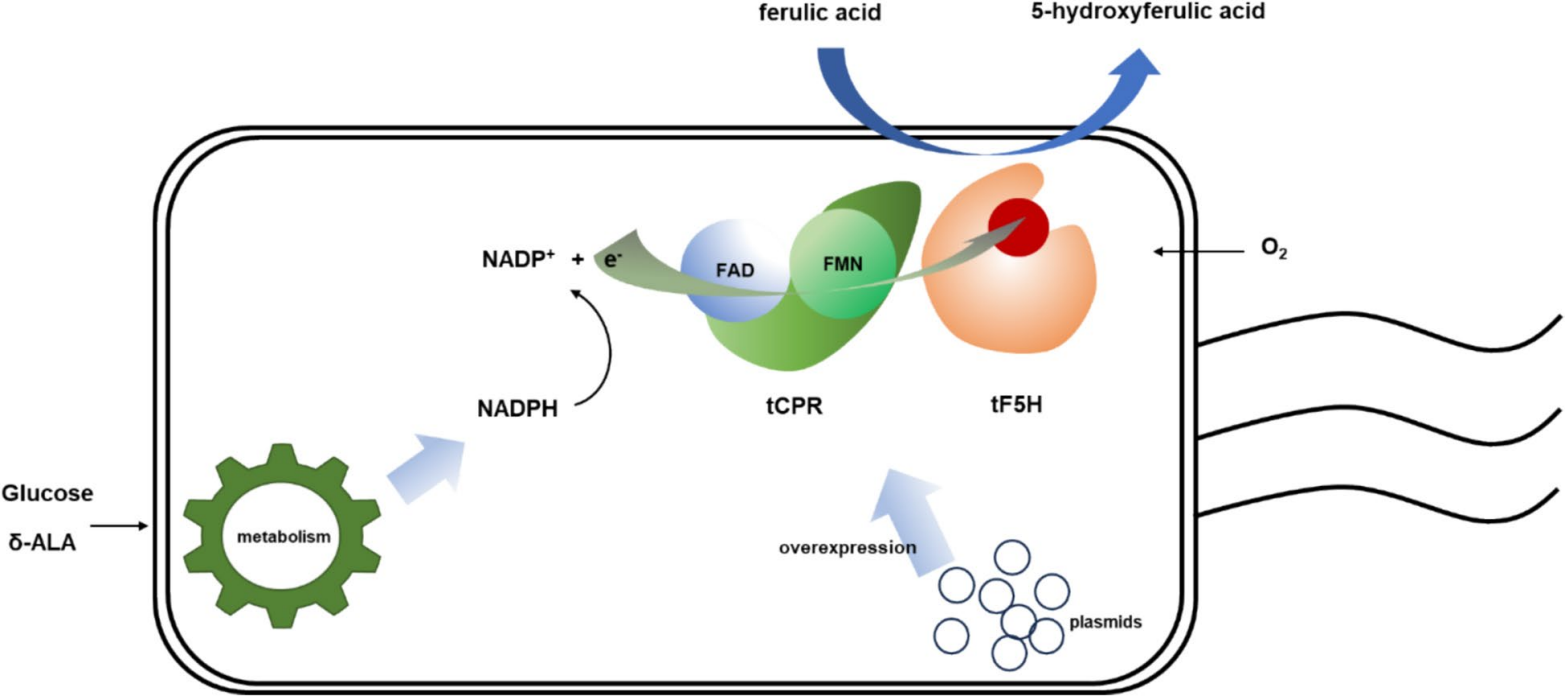
Schematic diagram of a catalytic system for heteroexpression of F5H and its redox partner protein in *E. coli*

Based on the type of cofactor P450s need for catalysis, F5H is categorized as a Class II heme-dependent P450 (Torres Pazmiño et al. 2010). The oxidative function of these Class II heme-dependent P450s which are mostly membrane proteins heavily relies on the presence of electron transfer partner proteins, such as CPRs, and various cofactors (Torres Pazmiño et al. 2010). To express these membrane proteins in *E. coli*, most of them should be truncated and co-expressed with a CPR (Zelasko et al. 2013; Hausjell et al. 2018). For instance, Nakagawa (Nakagawa et al. 2016) co-expressed SalSNcut and ATR2 in *E. coli* for the total biosynthesis of opiates. Lautier (Lautier et al. 2023) used cutCPR to supply electrons for CYP97H1, enabling *β*-Cryptoxanthin production in *E. coli.* Additionally, *E. coli* BL21(DE3) does not naturally consume ferulic acid efficiently (Deng et al. 2020). Therefore, we employed the CPR as a reducing partner for C-H bond activation of F5H in *E. coli*. As illustrated in Fig. 1, a strain which co-overexpressed the CPR and F5H was designed to realize the biofunctionability of F5H in vivo.

In Fig. 1, we have engineered a bacterial strain co-expressing cytochrome P450 reductase (CPR) and ferulate-5-hydroxylase (F5H) to facilitate the in vivo functionality of F5H. Concurrently, Fig. 1 depicts the predicted mechanism of F5H-mediated hydroxylation of ferulic acid, resulting in the production of 5-hydroxyferulic acid (5-HFA). This design aims to ensure efficient expression of F5H within the bacterial host while elucidating its specific role in the biosynthesis of 5-HFA.

**Fig. 2:**
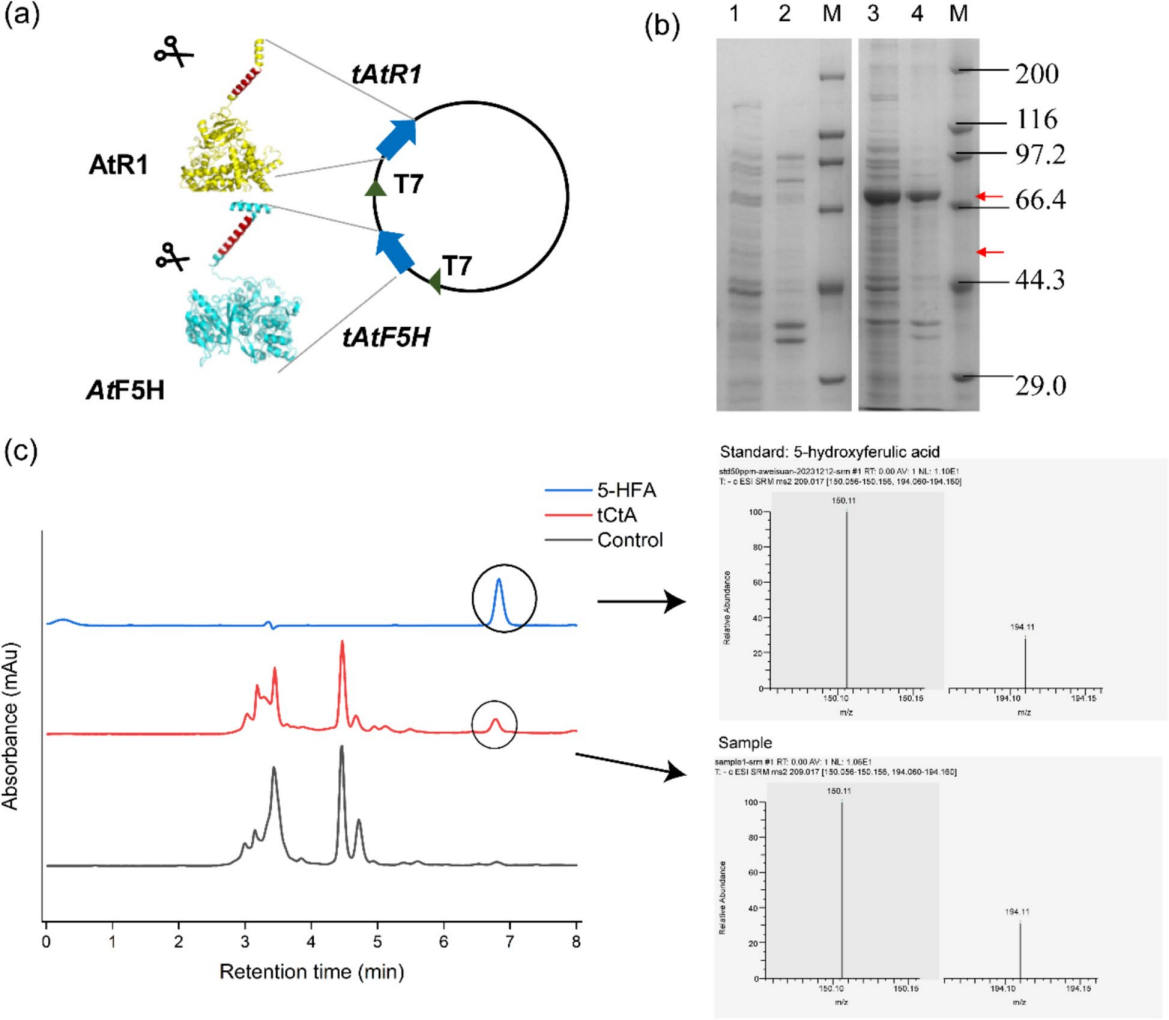
Biosynthesis and identification of 5-HFA. **a** Diagram of plasmid construction for the co-expression of truncated proteins tAtR1 and t*At*F5H (**b**) SDS-PAGE analysis of strains Control and tCtA (M: Protein Marker; 1: Supernatant of strain Control; 2: Precipitation of strain Control; 3: Supernatant of strain tCtA; 4: Precipitation of strain tCtA; The upper red arrow represents the truncated CPRs, while the lower red arrow indicates the truncated F5H); (**c**) HPLC chromatogram of fermentation broth(left); ESI-TOF–MS/MS spectrum of standard 5-HFA and the sample (right, 194.11 [M-H-CH_3_]^−^, 150.11 [M-H-C_2_H_3_O_2_]^−^)

We initially selected two proteins encoding F5Hs, derived from *A. thaliana* (*At*F5H, Uniprot ID: Q9C543), and *Aspergillus flavus* (*Af*F5H, Uniprot ID: B8NU02.1), as well as a protein encoding CPRs, the redox partner protein, *A. thaliana* (AtR1, Uniprot ID: Q9SB48) to construct strains for 5-HFA synthesis. In prior experiments, we observed that the overexpression of plant-derived *At*F5Hs with full-sequence in *E. coli* resulted in challenges in strain growth. Subsequently, we recognized that transmembrane regions of eukaryotic membrane proteins can lead to protein misfolding and aggregation in bacterial hosts, resulting in reduced growth rates and affecting protein yield and quality (Durairaj and Li 2022). Therefore, we uploaded these sequences to https://dtu.biolib.com/DeepTMHMM to predicted transmembrane regions. The results showed that three proteins were all have transmembrane regions (Supplementary Fig. S1). As shown in Table 2. *At*F5H has a single transmembrane region ranging from residues 15–32; *Af*F5H displayed multiple transmembrane regions, including residues 7–29, 176–198, 205–227, 232–254 and 291–313; and finally, AtR1 contains a transmembrane domain covering residues 27–46. Functional recombinant expression of eukaryotic microsomal CYPs in *E. coli* is generally considered challenging. We then chose *At*F5H and AtR1, which each only had one N-terminal transmembrane region. For their functional expression, *At*F5H and AtR1 were codon-optimized and their native N-terminal signal peptide sequences predicted were eliminated to result in truncated proteins t*At*F5H (a 2–32 amino acid deletion in *At*F5H; 55 KDa) and tAtR1 (a 2–46 amino acid deletion in AtR1; 72 KDa), respectively. Next, t*At*F5H was co-expressed with tAtR1 in *E. coli* BL21(DE3) to construct strain tCtA (Fig. 2a, Table 1). SDS-PAGE analysis showed that t*At*F5H was expressed at an extremely low level (Fig. 2b).

**Table 2:** Amino acid composition of the transmembrane helix

Enzyme	Transmembrane helix	Amino acid region	Amino acid sequence
*At*F5H	TM1	15–32	PTTALVIVVSLFIFIGFI
*Af*F5H	TM1	7–29	AQILVVSLGLLIFVLLCPWFGYL
	TM2	176–198	LTVALDSIIFGICLRLFFGVSLS
	TM3	205–227	YYVKIFTRVTGAMMLFVSQLVPW
	TM4	232–254	VVGIVAGFPIYYYWVRLIIYLYP
	TM5	291–313	VHISSLIVRLTLIVFLPVDVLIA
AtR1	TM1	27–46	VVLVIATTSLALVAGFVVLL

The constructed strains tCtA and Control (contain empty vector pETDuet-1) were grown in the medium containing ferulic acid to measure the production of 5-HFA by t*At*F5H. According to HPLC analysis (Fig. 2c), 5-HFA was not detected in the fermentation broth of the control strain. However, the product 5-HFA was indeed identified in the fermentation broth of strains tCtA based on comparison of mass spectrometry fragmentation patterns with the 5-HFA standards. The tandem mass spectrometry (MS/MS) fragment pattern of the peak (catalyzed by strains tCtA) was almost identical to that of 5-HFA (Fig. 2c), indicating that t*At*F5H had ferulate-5-hydroxylase (F5H) activity in *E. coli*. Thus, we decided to use t*At*F5H for the biosynthesis of 5-HFA.

### Coupling *At*F5H and AtR1 in *E.coli* for enhanced biosynthesis of 5-HFA

**Fig. 3:**
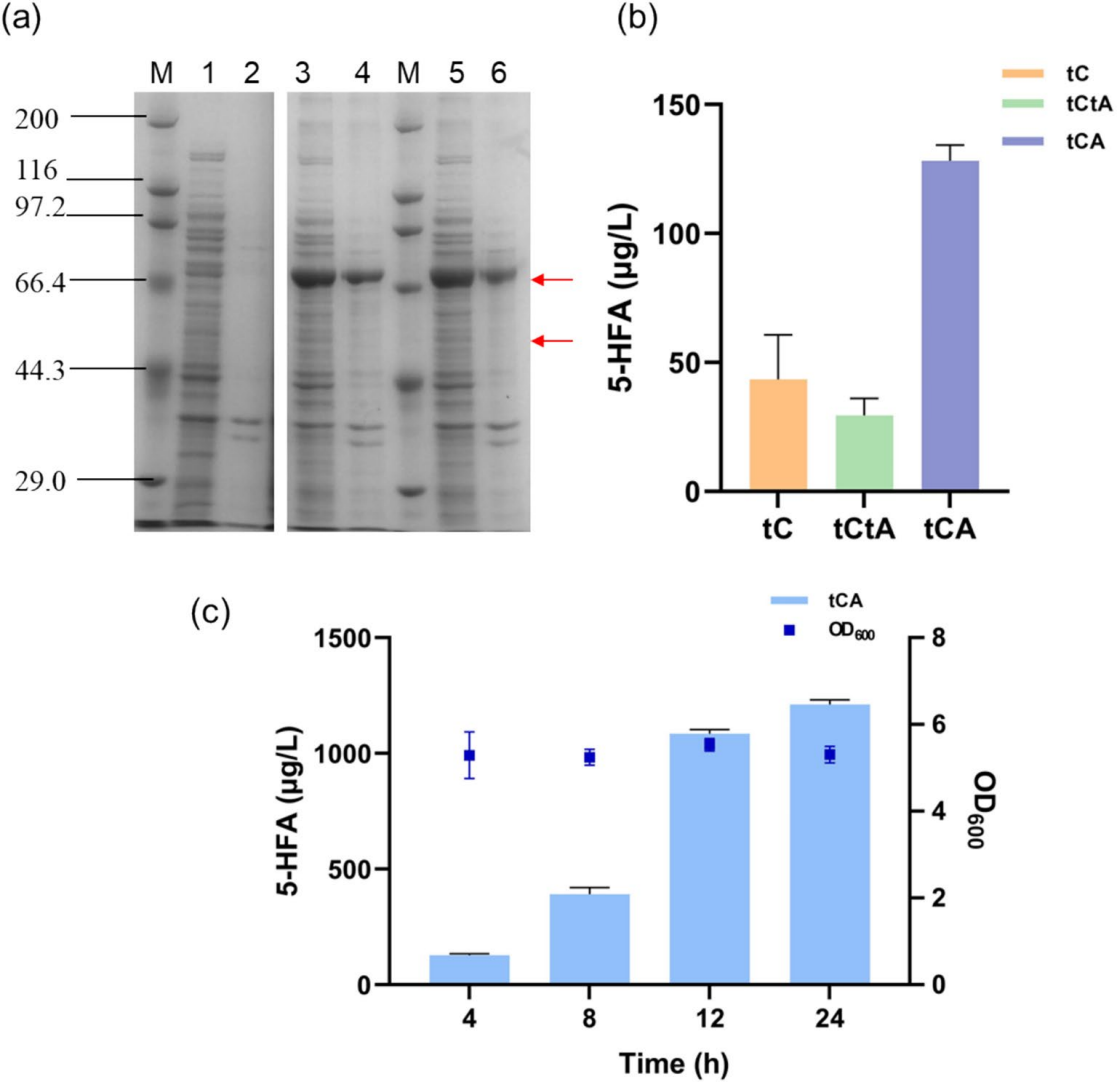
Bioconversion of ferulic acid by recombinant strains (tC, tCtA, tCA). (**a**) SDS-PAGE analysis of strains tC, tCtA and tCA (M: Protein Marker; 1: Supernatant of strain tC; 2: Precipitation of strain tC; 3: Supernatant of strain tCtA; 4: Precipitation of strain tCtA; 5: Supernatant of strain tCA; 6: Precipitation of strain tCA; The upper red arrow represents two different modifications of the AtR1 enzyme, while the lower red arrow indicates the truncated *At*F5H); (**b**) The concentration of 5-HFA in the fermentation broth broth produced by strains tC, tCtA and tCA (4 h); (**c**) The concentration of 5-HFA synthesized by strain tCA

Previous studies (Barnes et al. 1991; Halkier et al. 1995) have demonstrated that substituting the second amino acid in P450 with alanine (“A”) significantly enhances protein expression in *E. coli*. Consequently, beyond merely truncating the N-terminal 2–46 residues of AtR1 to generate the truncated protein tAtR1, an additional alanine residue was introduced post-deletion to create the modified truncated protein tAtR1_mod_. This protein, along with the truncated t*At*F5H, was co-expressed in *E. coli*, resulting in the recombinant strain tCA. Additionally, we also constructed another strain, tC, which expresses only the truncated t*At*F5H (Fig. 3a & Fig.S2). These recombinant strains—tC, tCtA, and tCA—were subsequently employed in the hydroxylation of ferulic acid, with the quantification of 5-HFA in the fermentation broth conducted (Fig. 3b). The results revealed that the fermentation broth of strain tCA contained 128 µg/L of 5-HFA, approximately fourfold higher than that produced by the lowest-yielding strain tCtA. This outcome underscores the effectiveness of the alanine addition at the N-terminus of the truncated membrane protein in enhancing the whole-cell catalytic efficiency. Moreover, the fermentation broth of strain tC, which solely expressed t*At*F5H, also exhibited a corresponding peak, despite the absence of CPR in *E. coli*. This observation suggests the presence of alternative enzymes or enzyme systems within *E. coli* capable of forming an electron transfer chain with F5H, thus providing the requisite cofactors for the oxidation required during hydroxylation. Under identical conditions, strain tCA synthesized a greater amount of 5-HFA. Specifically, strain tCA catalyzed the conversion of 0.1 g/L of ferulic acid to 128 µg/L of 5-HFA within 4 h, and reached 1086 µg/L after 12 h (Fig. 3c).

### Scaffold assisted electronic channel strategy for biosynthesis of 5-HFA by *E.coli*

**Fig.4:**
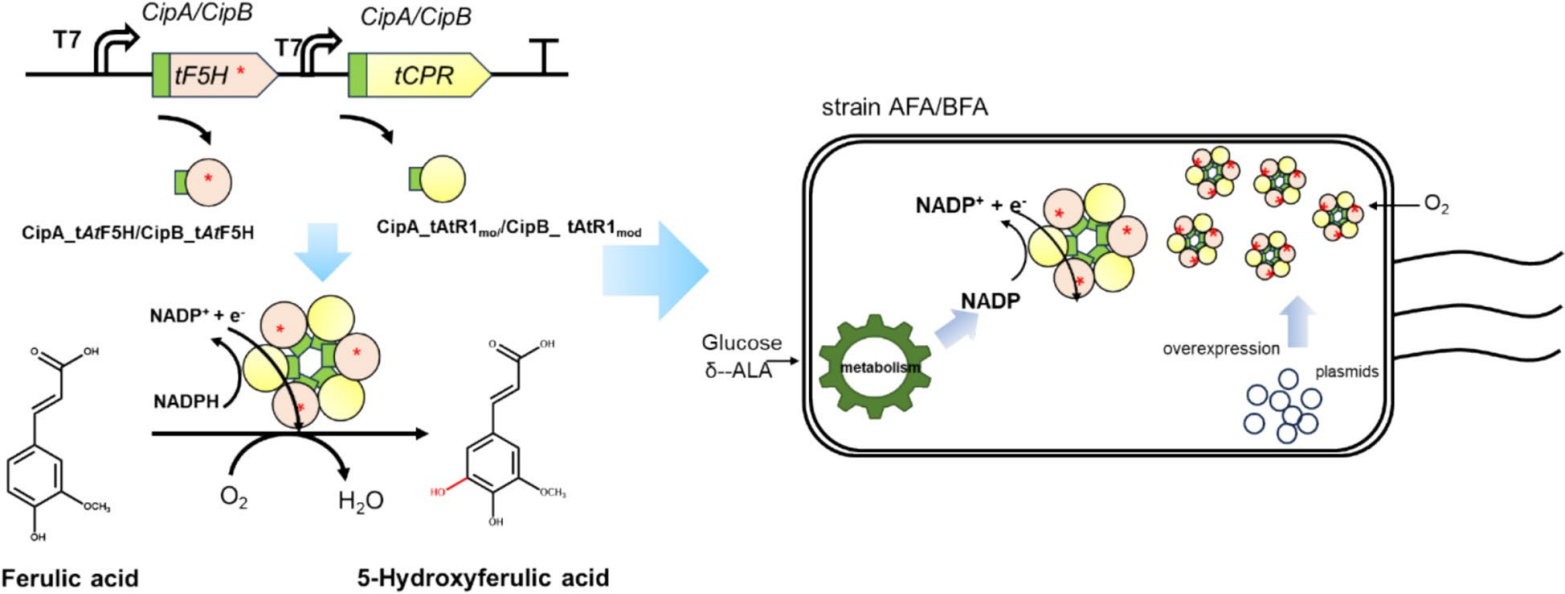
Design, characterization and application of the scaffold proteins assisted electronic channel strategy

Apart from the challenges associated with heterologous expression of eukaryotic cytochrome P450s, the low efficiency of electron transfer reactions between P450 and its partner protein is another significant hurdle for the application of P450s in the biosynthesis of natural products. It was also hypothesized that the spatial distance of the P450s and CPRs might leads to inefficient electron transfer between them in this work. Many strategies, such as fusion and self-assembly, have been extensively employed to improve the efficiency of electron transfer. Here, we designed a self-assembly strategy to enhance the electron channeling, by tethering tF5H and tAtR1_mod_ together using a scaffold protein (Fig. 4a). CipA and CipB derived from *Photorhabdus luminescens* can serve as scaffolds to effectively incorporate exogenous proteins into protein crystalline inclusions (PCIs)(Wang et al. 2017). In this work, the CipA or CipB scaffold proteins were directly fused to the N-terminal of t*At*F5H and tAtR1_mod_ without any linkers for electron channeling in *E. coli*, resulting in strains AFA and BFA, respectively*.* They were used for bioconversion of ferulic acid.

**Fig.5:**
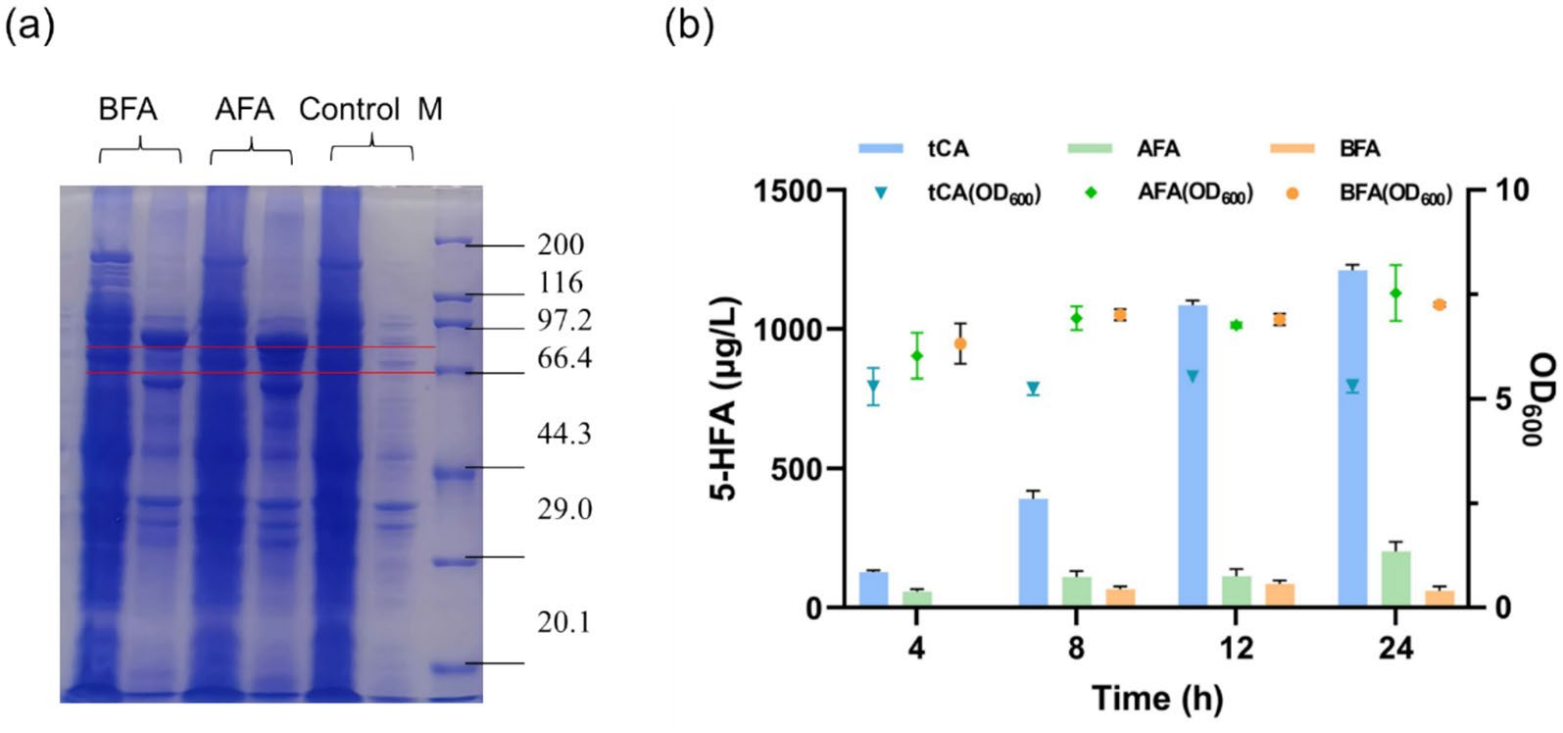
The biosynthesis of 5-HFA using recombinant strains. **a** SDS-PAGE analysis of strains BFA, AFA, and Control (M: Protein Marker; Left: Supernatant fractions; Right: Precipitation fractions); (**b**) The concentration of 5-HFA in the fermentation broth using strains tCA, AFA and BFA (12 h). Data displayed as mean ± s.d. (*n* = 3)

Compared to the strain tCA expressing truncated F5H and CPR without scaffold proteins, the growth (OD_600_) and catalytic ability to synthesize 5-HFA in strains AFA and BFA, which were obtained by adding self-assembling peptides to the truncated F5H and CPR, were significantly reduced regardless of whether the scaffold protein was CipA or CipB from 4 to 24 h (Fig. 5b). The growth of strain AFA, expressing enzymes with the transmembrane region replaced by CipA, is nearly identical to that of the strain BFA containing CipB, maintaining an OD_600_ of approximately 5–7. Between 4 and 12 h, the production of 5-HFA by strain AFA through the biotransformation of ferulic acid consistently remains slightly higher than that of strain BFA. The productions of 5-HFA obtained from strains AFA and BFA through the biotransformation of ferulic acid continue to increase, culminating in peak concentrations of 203.7 µg/L at 24 h and 85.8 µg/L at 12 h, respectively. However, strain tCA consistently led in the synthesis of 5-HFA, producing approximately 1211.6 µg/L at 24 h, which is 19.9 times higher than strain BFA’s production of 61.0 µg/L, and about 5.9 times the production of strain AFA. The results indicate that the addition of Cip scaffold proteins either CipA or CipB did not enhance the production of 5-HFA in vivo in this study. It differs from the findings of Park (Park et al. 2022) et al., who effectively enhanced the production of lutein, (+)-nootkatone, apigenin and L-DOPA by applying the CipB scaffold-assisted electron channeling strategy. Although SDS-PAGE confirmed high expression levels (Fig. 5a), the scaffold protein did not enhance production due to several potential factors. These include improper enzyme alignment within the scaffold, which could hinder efficient substrate channeling. Additionally, the introduction of the scaffold may impose a metabolic burden on the host cells, diverting resources from 5-HFA synthesis. Furthermore, issues such as misfolding and aggregation of the scaffold protein itself could impair its intended function.

### N-terminal modifications of *At*F5H for biosynthesis of 5-HFA by *E.coli*

**Fig. 6:**
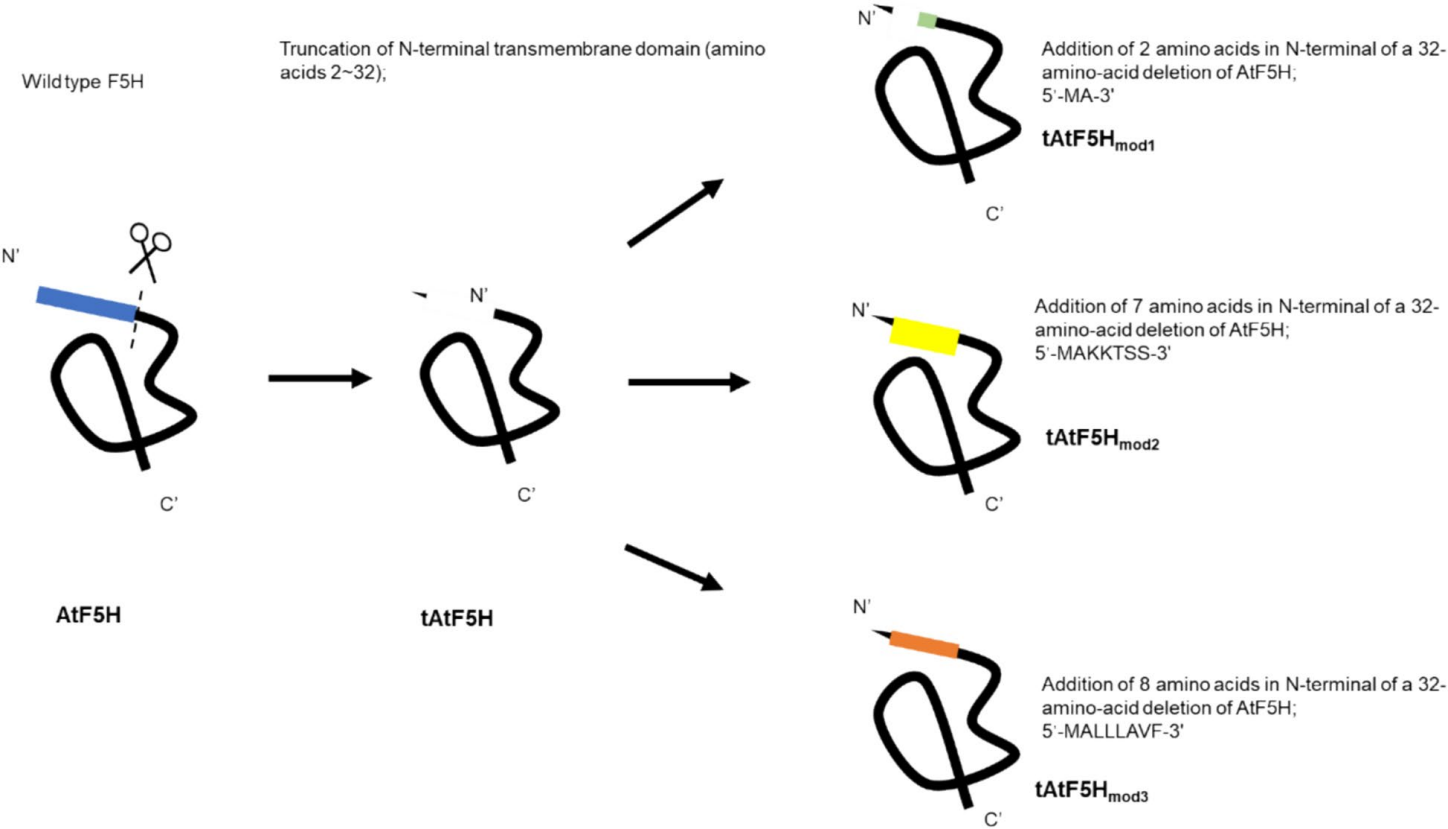
Schematic diagram of N-terminal modification of F5H (*Arabidopsis* F5H_mod_)

Most eukaryotic P450s are membrane proteins and usually have transmembrane helixes for anchoring. Expressing these membrane-bound proteins in *E. coli* often lead to challenges for the enzyme inactivity. The issues are frequently addressed by performing modifications at the N-terminus of P450s (Zelasko et al. 2013). Halkier demonstrated that altering the second codon of CYP450 cDNA from TGG to GCT significantly enhances P450 expression levels in *E. coli* (Halkier et al. 1995). Similarly, Barne et al. improved the expression of P450 17α-hydroxylase (CYP17A1) by modifying its N-terminal sequence from “MWLLLAVF” to “MALLLAVF”, which enhanced ribosomal recognition and translation initiation efficiency (Barnes et al. 1991). Furthermore, replacing “LLLAVF” with “AKKTSS” was found to maintain membrane localization while increasing protein expression levels (Zelasko et al. 2013). Hence, we deleted the transmembrane regions from both *At*F5H and AtR1, which resulted in a 32-amino-acid deletion of *At*F5H and a 46-amino-acid deletion of AtR1, respectively. Then, we added the amino acid sequence “MA” and more hydrophilic peptide sequences such as “MAKKTSS”, or a hydrophobic peptide sequence “MALLLAVF” in the N-terminally-truncated *At*F5H(where amino acids 1–32 were deleted) and co-overexpressed with tAtR1, resulting in strain 1CA, 2CA and 3CA (Fig. 6). They were all used for 5-HFA production.

**Fig. 7:**
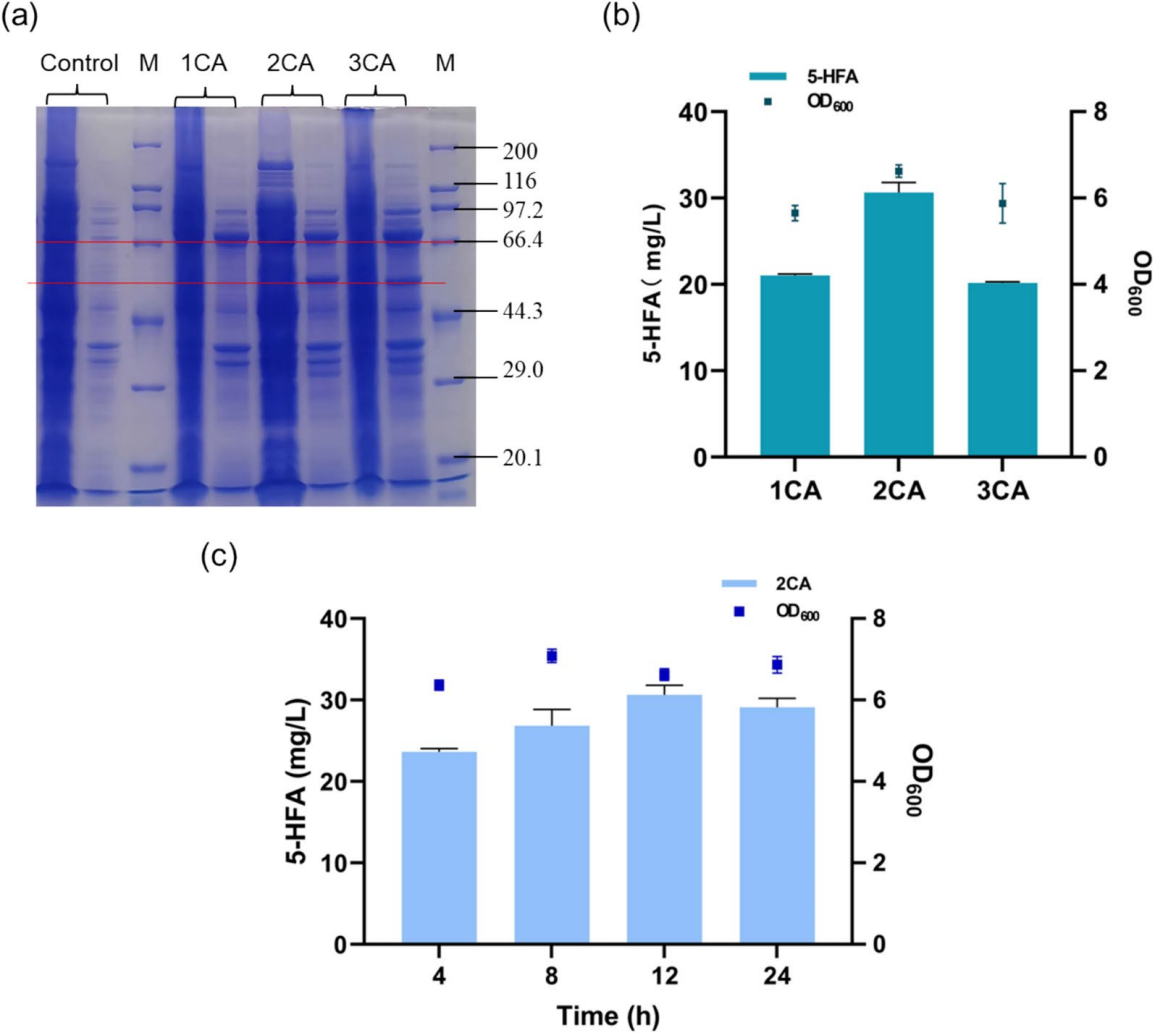
Biosynthesis of 5-HFA by biotransformation (Strains 1CA, 2CA, 3CA). (**a**) SDS-PAGE analysis of the heterologous expression of recombinants with various N-terminal modifications of F5H (supernatant on the left and the precipitates on the right); (**b**) Concentration of 5-HFA synthesized by strains 1CA, 2CA and 3CA with different F5Hs_mod_ (12 h), and (**c**) concentration of 5-HFA synthesized by strain 2CA. Data displayed as mean ± s.d. (n = 3)

Notably, strain 2CA showed best among three strains in the accumulation of 5-hydroxyferulic acid (Fig. 7b), achieving 30.6 mg/L. By modifying the N-terminus of F5H, strain 2CA exhibited significantly better growth and yield compared to strains 1CA and 3CA. Strain 3CA had the lowest 5-HFA production of 20.2 mg/L, which is only 66% of strain 2CA. SDS-PAGE analysis revealed inconsistent expression of t*At*F5H with different N-terminal modifications in *E. coli*. The majority of inclusion bodies were observed for F5H_mod_ with “MAKKTSS” or “MALLLAVF”, except for F5H_mod_ with “MA” (Fig. 7a & Fig.S2). Strain 1CA expressing “MA”-tagged F5H_mod_ produced only 21 mg/L of 5-HFA, slightly higher than strain 3CA (Fig. 7b). By removing F5H’s transmembrane region and replacing it with “MAKKTSS”, we obtained the 2CA strain, which effectively facilitated the synthesis of 5-HFA. The concentration of 5-HFA in strain 2CA gradually increased, reaching a peak at 12 h (Fig. 7c) 2–12, showcasing the effectiveness of this modification.

### Extraction of ferulic acid from agricultural by-products for 5-HFA synthesis

**Fig.8:**
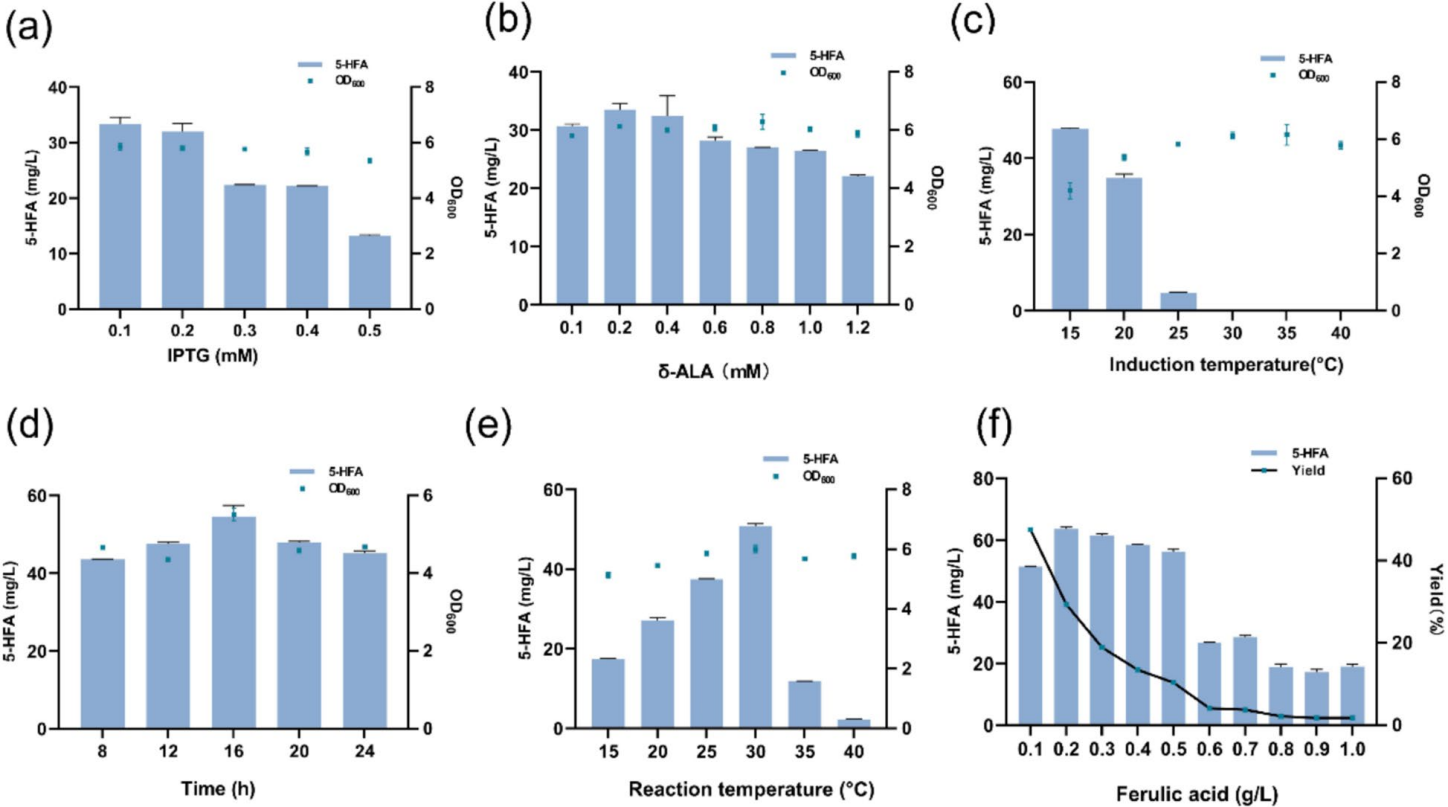
Optimization of fermentation conditions on 5-HFA synthesis with strain 2CA The optimized parameters (**a**–**f**) include. (**a**) concentration of δ-ALA, (**b**) concentration of IPTG, (**c**) induction temperature, (**d**) Substrate Addition Timing, (**e**) Reaction temperature, and (**f**) concentration of ferulic acid. Data displayed as mean ± s.d. (n = 3)

The engineered strain 2CA above has shown significant advantages in synthesizing 5-HFA. To further enhance its efficiency in biotransforming ferulic acid, we are optimizing the fermentation conditions focuses on fine-tuning various parameters to maximize the concentration of 5-HFA (Fig. 8). Key factors such as IPTG concentration, δ-ALA concentration, induction temperature, substrate addition timing, biotransformation temperature and substrate concentration play critical roles in maximizing production of 5-HFA. As shown in Fig. 8a, at 0.1 mM IPTG, the product was slightly higher than at 0.2 mM, reaching a maximum concentration. As IPTG concentration increased, the 5-HFA concentration decreased. These results indicate that 0.1 mM IPTG is sufficient for recombinant protein expression. As a heme-dependent monooxygenase, F5H requires the exogenously supplied precursor δ-ALA due to *E. coli*’s inability to synthesize heme. Figure 8b shows optimal δ-ALA concentration at 0.2 mM, with 5-HFA concentrations peaking at this level and diminishing significantly at 1.2 mM. Induction temperature significantly affects growth and protein expression in recombinant *E. coli.* The optimal induction temperature for stable protein expression is established at 15 °C (Fig. 8c). While optimal growth is observed at 35 °C, close to the species’ ideal temperature of 37 °C, concentrations of 5-HFA sharply decline and become undetectable above 30 °C, suggesting potential protein misfolding and inactivation. The timing of ferulic acid addition critically affects its conversion, with peak 5-HFA production at 16 h post-addition with 54.6 mg/L, 1.25 times that at 8 h. (Fig. 8d). Biotransformation temperature crucially influences 5-HFA yield, with peak production at 30 °C, nearly 22 times higher than at 40 °C, indicating a sharp decline in yield at higher temperatures (Fig. 8e).

Overall, under optimal conditions—0.2 mM δ-ALA, 0.1 mM IPTG, 15 °C induction temperature, and biotransformation at 30 °C after adding ferulic acid at 16 h—strain 2CA produced 50.8 mg/L of 5-HFA after 12 h, with the highest conversion rate of 47%. Substrate concentration studies revealed a peak 5-HFA concentration of 63.6 mg/L at 0.2 g/L substrate concentration, which conversion rate was 27 times than that at 1 g/L. (Fig. 8f). Heterologous expression of plant-derived P450 enzymes in *E. coli* yields low amounts, making it challenging to find substitutes quickly, though yeast or *E. coli* expression offers versatility, especially for complex natural products requiring specific P450s (Furuya and Kino 2014; Chu et al. 2017; Sun et al. 2024). This comprehensive approach to fine-tuning each parameter not only maximizes the production of 5-HFA but also provides a robust framework for future scale-up and industrial applications. To enhance the substrate tolerance of the system (0.2 g/L), future experiments will focus on selecting strains with increased substrate tolerance through adaptive laboratory evolution. Additionally, we will employ site-directed mutagenesis to improve the hydroxylation efficiency of the key enzyme F5H. We believe these strategies will significantly enhance the practical applicability of the system.

**Fig. 9:**
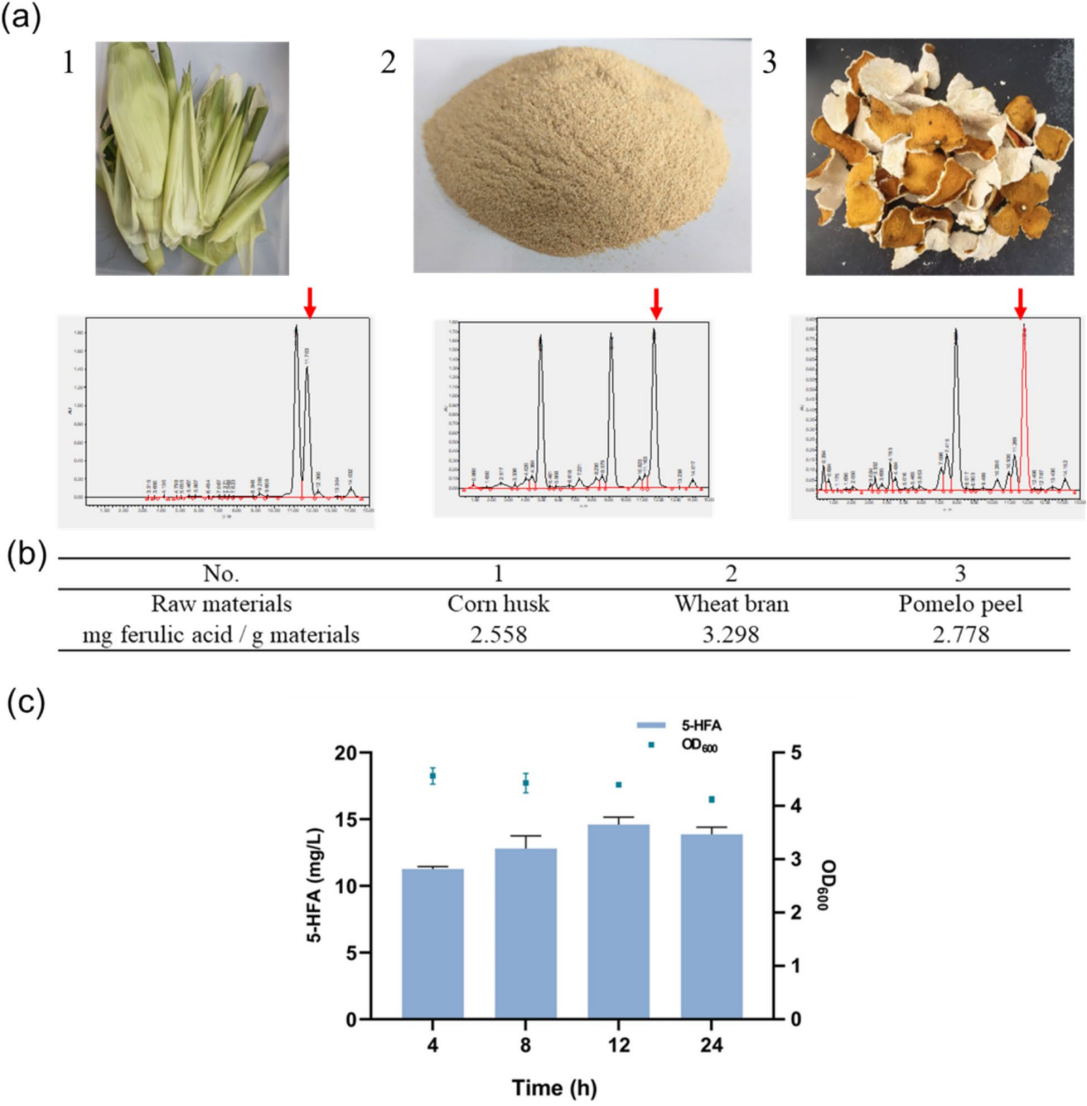
Extraction of ferulic acid from agricultural by-products for the synthesis of 5-HFA. a Raw materials for extracting ferulic acid and HPLC chromatogram of the extract solution (red arrow refers to the peak of ferulic acid); (**b**) Concentration of ferulic acid extracted from raw materials; (**c**) Concentration of 5-HFA synthesized by strain 2CA. Data displayed as mean ± s.d. (n = 3)

Refined ferulic acid is costly for use as a substrate in 5-HFA production. Therefore, identifying a cost-effective alternative substrate is economically viable. China consumes a large number of crops annually, resulting in numerous agricultural by-products, such as grain husks and fruit peels. These by-products contain significant energy and abundant natural resources that can be reused. Currently, many agricultural by-products have been found to contain rich phenolic compounds like ferulic acid, which can be turned into valuable resources for extracting high-value natural products, maximizing waste utilization. Materials for extracting ferulic acid on the left are corn husks, wheat bran, and pomelo peels, from top to bottom (Fig. 9a–c). The ferulic acid content is indicated by the red arrow in Fig. 9a. According to Fig. 9b, the ferulic acid content was highest in wheat bran at 3.298 mg ferulic acid /g materials, followed by pomelo peels, with corn husks having the lowest content at 2.558 mg ferulic acid /g materials. Then, ferulic acid crude extract from wheat bran was directly added to the fermentation broth of recombinant strain 2CA at a concentration of 0.1 g/L. As shown in Fig. 9c, the production of 5-HFA gradually increased, reaching a peak concentration of 14.6 mg/L at 12 h, corresponding to a yield of 0.48 mg/g wheat bran. This concentration was lower than that achieved with high-purity ferulic acid, suggesting that the crude ferulic acid extract was unrefined, with residual organic materials and high impurity content. These factors likely impacted the activity of the sensitive P450 enzymes involved. It demonstrates the potential for utilizing agricultural waste as a substrate for the biotransformation by engineered strains to synthesize high-value antioxidant 5-HFA, highlighting its application in bioresource utilization.

## Conclusion

In this work, we co-expressed eukaryotic *At*F5H and CPR in *E. coli* for 5-HFA synthesis*.* It also demonstrated the application of an engineered a bacterial strain co-expressing truncated CPR and F5H for biocatalysis through simple fermentation. We explored N-terminal modification approaches to enhance the biotransformation efficiency of engineered strains. Our results revealed that strain 2CA, with the hydrophilic sequence “MAKKTSS” at the truncated *At*F5H N-terminus and “MA” at the truncated AtR1 N-terminus, promoted the *ortho*-hydroxylation of ferulic acid, achieving a 5-HFA concentration of 63.6 mg/L. When using agricultural waste as the substrate, the yield of 5-HFA was 0.48 mg/g wheat bran. This study not only provides a valuable reference for the expression of plant-derived P450s in *E. coli* but also highlights the potential of these engineered strains to effectively utilize biological resources while offering economic benefits.

## Supplementary Information

Additional file 1.

## Data Availability

All data that support the findings of this study are included within this paper.

## Abbreviations

F5H: Ferulate-5-hydroxylase
5-HFA: 5-Hydroxyferulic acid
CPR: NADPH-cytochrome P450 reductase
δ-ALA: δ-Aminolevulinic acid
