# Supplementary material for: Bioactive expression of eukaryotic cytochrome P450 ferulate-5-hydroxylase in Escherichia coli for sustainable synthesis of antioxidant 5-hydroxyferulic acid
Source: Bioresour Bioprocess. 2025 Jul 15;12(1):75. doi: 10.1186/s40643-025-00919-z (PMC12263503; doi:10.1186/s40643-025-00919-z)
Supplement: Supplementary file 1 — Additional file 1. [file 40643_2025_919_MOESM1_ESM.docx]

**Supplementary materials**

**Bioactive Expression of Eukaryotic Cytochrome P450 Ferulate-5-Hydroxylase in *Escherichia coli* for Sustainable Synthesis of Antioxidant 5-Hydroxyferulic Acid**

Ping Sun^1^, Yuan Tian ^1^, Luyi Wang^1^, Pengcheng Chen^1^, Dan Wu^1^, Pu Zheng^1^*

^1^ School of Biotechnology and Key Laboratory of Industrial Biotechnology of Ministry of Education, Jiangnan University, Wuxi 214122, China

^*^ Corresponding author

E-mail: zhengpu@jiangnan.edu.cn

Tel./Fax: +86-510-85918156

**Supplementary materials**

Table S1. Primers used in this work.

Table S2. Amino acid sequences of the redox partners (CPRs), the cytochromes P450 (F5Hs) and scaffold proteins.

Figure S1. Prediction of transmembrane regions for membrane proteins ATR1, *At*F5H, and *Af*F5H.

Figure S2. Western Blot (WB) analysis of *At*F5H_mod_s.

Table S1. Primers used in this work.

| **Protein** | |  | **Forward primer (5’-3’)** | **Reverse primer (5’-3’)** |
| --- | --- | --- | --- | --- |
| tAtR1 |  | | GGAATTCCATATGTGGAAAAAAACCACCGCGG | CCGCTCGAGTTACCACACATCGCGCAGATAGC |
| tAtR1_mod_ | tAtR1_mod_ (“MA”-tagged) | | GGAATTCCATATGGCATGGAAAAAAACCACCGCG | CCGCTCGAGTTACCACACATCGCGCAGATAGC |
| tAtF5H |  | | AGAAGGAGATATACCATGACCCGCCGTCGCC | AGGCGCGCCGAGCTCGAATTCTTACAGCGCGCAAATCAGGCGGG |
| tAtF5H_mod_ | tAtF5H_mod1_ (“MA”-tagged) | | CATGCCATGGCAACCCGCCGTCGCCGC | GGAATTCTTACAGCGCGCAAATCAGG |
|  | tAtF5H_mod2_ (“MAKKTSS”-tagged) | | CATGCCATGGCGAAGAAAACCTCTTCTACCCGCCGTCGCCGC | GGAATTCTTACAGCGCGCAAATCAGG |
|  | tAtF5H_mod2_ (“MALLLAVF”-tagged) | | CATGCCATGGCTCTGTTATTAGCAGTTTTTACCCGCCGTCGCCGCCCGCCGTATC | GGAATTCTTACAGCGCGCAAATCAGG |
| CipA_tAtF5H | CipA | | AGAAGGAGATATACCATGATTAACGATATGCATCCGAGC | CATGCTAATTTCCACGCAG |
|  | tAtF5H | | GTGGAAATTAGCATGACCCGCCGTCGCCGC | GCCGAGCTCGAATTCTTACAGCGCGCAAATCAGG |
| CipA_tAtR1_mod_ | CipA | | GAAGGAGATATACATATGATTAACGATATGCATCCGAGC | CATGCTAATTTCCACGCAG |
|  | tAtR1_mod_ | | GTGGAAATTAGCATGGCATGGAAAAAAACCACCG | TTTACCAGACTCGAGTTACCACACATCGCGCAG |
| CipB_tAtF5H | CipB | | AGAAGGAGATATACCATGATTATTAAAAAAGATATTCTGC | AATTTCCACGCCCACAATAT |
|  | tAtF5H | | GTGGGCGTGGAAATTACCCGCCGTCGCCGC | GCCGAGCTCGAATTCTTACAGCGCGCAAATCAGG |
| CipB_tAtR1_mod_ | CipB | | GAAGGAGATATACATATGATTATTAAAAAAGATATTCTGC | AATTTCCACGCCCACAATAT |
|  | tAtR1_mod_ | | GTGGGCGTGGAAATTGCATGGAAAAAAACCACCG | TTTACCAGACTCGAG TTACCACACATCGCGCAG |

Note: The underlined regions represent enzyme cleavage sites.

**Table S2**. Amino acid sequences of the redox partners (CPRs), cytochromes P450 (F5Hs), scaffold proteins and the N-terminal modified proteins .

| Name | Amino acid sequences |
| --- | --- |
| AtR1 | >sp\|Q9SB48\|NCPR1_ARATH NADPH--cytochrome P450 reductase 1 OS=Arabidopsis thaliana OX=3702 GN=ATR1 PE=1 SV=1  MTSALYASDLFKQLKSIMGTDSLSDDVVLVIATTSLALVAGFVVLLWKKTTADRSGELKPLMIPKSLMAKDEDDDLDLGSGKTRVSIFFGTQTGTAEGFAKALSEEIKARYEKAAVKVIDLDDYAADDDQYEEKLKKETLAFFCVATYGDGEPTDNAARFYKWFTEENERDIKLQQLAYGVFALGNRQYEHFNKIGIVLDEELCKKGAKRLIEVGLGDDDQSIEDDFNAWKESLWSELDKLLKDEDDKSVATPYTAVIPEYRVVTHDPRFTTQKSMESNVANGNTTIDIHHPCRVDVAVQKELHTHESDRSCIHLEFDISRTGITYETGDHVGVYAENHVEIVEEAGKLLGHSLDLVFSIHADKEDGSPLESAVPPPFPGPCTLGTGLARYADLLNPPRKSALVALAAYATEPSEAEKLKHLTSPDGKDEYSQWIVASQRSLLEVMAAFPSAKPPLGVFFAAIAPRLQPRYYSISSSPRLAPSRVHVTSALVYGPTPTGRIHKGVCSTWMKNAVPAEKSHECSGAPIFIRASNFKLPSNPSTPIVMVGPGTGLAPFRGFLQERMALKEDGEELGSSLLFFGCRNRQMDFIYEDELNNFVDQGVISELIMAFSREGAQKEYVQHKMMEKAAQVWDLIKEEGYLYVCGDAKGMARDVHRTLHTIVQEQEGVSSSEAEAIVKKLQTEGRYLRDVW |
| *At*F5H | >tr\|Q9C543\|Q9C543_ARATH Ferulate-5-hydroxylase OS=Arabidopsis thaliana OX=3702 GN=fah1 PE=3 SV=1  MESSISQTLSKLSDPTTALVIVVSLFIFIGFITRRRRPPYPPGPRGWPIIGNMLMMDQLTHRGLANLAKKYGGLCHLRMGFLHMYAVSSPEVARQVLQVQDSVFSNRPATIAISYLTYDRADMAFAHYGPFWRQMRKVCVMKVFSRKRAESWASVRDEVDKMVRSVSCNVGKTINVGEQIFALTRNITYRAAFGSACEKGQDEFIRILQEFSKLFGAFNVADFIPYFGWIDPQGINKRLVKARNDLDGFIDDIIDEHMKKKENQNAVDDGDVVDTDMVDDLLAFYSEEAKLVSETADLQNSIKLTRDNIKAIIMDVMFGGTETVASAIEWALTELLRSPEDLKRVQQELAEVVGLDRRVEESDIEKLTYLKCTLKETLRMHPPIPLLLHETAEDTSIDGFFIPKKSRVMINAFAIGRDPTSWTDPDTFRPSRFLEPGVPDFKGSNFEFIPFGSGRRSCPGMQLGLYALDLAVAHILHCFTWKLPDGMKPSELDMNDVFGLTAPKATRLFAVPTTRLICAL |
| *Af*F5H | >sp\|B8NU02.1\|LNAD_ASPFN RecName: Full=Cytochrome P450 monooxygenase lnaD; AltName: Full=Ferulate-5-hydroxylase; AltName: Full=Lna diastereomeric piperazines biosynthesis cluster protein D  MAASTTAQILVVSLGLLIFVLLCPWFGYLRLPSSMRWWPSIPSGPLSALRLSLKEYSGSRSSEDGYKAFSKKAELFAICNPSFYPQVLLPPEQIPWLLSQPENVLSHEKANEDVHALPFLAPAFDNYDHLELIRAIRTDLTRNIPNTEDAFLDELRHTTNEVLGAPGDNAWKEVNLTVALDSIIFGICLRLFFGVSLSRNRTFVYYVKIFTRVTGAMMLFVSQLVPWPLKPVVGIVAGFPIYYYWVRLIIYLYPTFKERIQCLRTKKETPPADMVTWMVDLAISQNPTRKVHISSLIVRLTLIVFLPVDVLIAMTDNFFLDLLSSDPDRKYYNALRQEAEAAFTNRDKTQPISQSMPYMESTIRESLRLSPLSDRMLSRRVVHKGGITLPDGQFLPRGTWLAVAAVGVHRDERTYEDPDEYRPFRFLSEDTETKEAKAMLVPVTSEKFLAFGHGRHSCPGRWFAAHAMKLIIGYILVNYDIEPLEKRPVNSVVGQTIIPQLDVKIRVRRRE |
| CipA | MINDMHPSLIKDKDMMDDVMLRSCKIIAMKIMPDKVMQVMVTVLMLDGTSEEMLLKWNLLDNRGMAIYKVLMEALCGKKDVKIGTVGKVGPLGCDYINCVEISM |
| CipB | MIIKKDILLHEDLIVDDELKVGKVEKVNIDILSPSSVIVSLNILGVVDDFHLLLVDDKDKDKIVLLYLSLLRVLHEKLDVKVKVAKSNLTKMKYIVGVEI |
| tAtR1 | MWKKTTADRSGELKPLMIPKSLMAKDEDDDLDLGSGKTRVSIFFGTQTGTAEGFAKALSEEIKARYEKAAVKVIDLDDYAADDDQYEEKLKKETLAFFCVATYGDGEPTDNAARFYKWFTEENERDIKLQQLAYGVFALGNRQYEHFNKIGIVLDEELCKKGAKRLIEVGLGDDDQSIEDDFNAWKESLWSELDKLLKDEDDKSVATPYTAVIPEYRVVTHDPRFTTQKSMESNVANGNTTIDIHHPCRVDVAVQKELHTHESDRSCIHLEFDISRTGITYETGDHVGVYAENHVEIVEEAGKLLGHSLDLVFSIHADKEDGSPLESAVPPPFPGPCTLGTGLARYADLLNPPRKSALVALAAYATEPSEAEKLKHLTSPDGKDEYSQWIVASQRSLLEVMAAFPSAKPPLGVFFAAIAPRLQPRYYSISSSPRLAPSRVHVTSALVYGPTPTGRIHKGVCSTWMKNAVPAEKSHECSGAPIFIRASNFKLPSNPSTPIVMVGPGTGLAPFRGFLQERMALKEDGEELGSSLLFFGCRNRQMDFIYEDELNNFVDQGVISELIMAFSREGAQKEYVQHKMMEKAAQVWDLIKEEGYLYVCGDAKGMARDVHRTLHTIVQEQEGVSSSEAEAIVKKLQTEGRYLRDVW |
| tAtR1_mod_ | MAWKKTTADRSGELKPLMIPKSLMAKDEDDDLDLGSGKTRVSIFFGTQTGTAEGFAKALSEEIKARYEKAAVKVIDLDDYAADDDQYEEKLKKETLAFFCVATYGDGEPTDNAARFYKWFTEENERDIKLQQLAYGVFALGNRQYEHFNKIGIVLDEELCKKGAKRLIEVGLGDDDQSIEDDFNAWKESLWSELDKLLKDEDDKSVATPYTAVIPEYRVVTHDPRFTTQKSMESNVANGNTTIDIHHPCRVDVAVQKELHTHESDRSCIHLEFDISRTGITYETGDHVGVYAENHVEIVEEAGKLLGHSLDLVFSIHADKEDGSPLESAVPPPFPGPCTLGTGLARYADLLNPPRKSALVALAAYATEPSEAEKLKHLTSPDGKDEYSQWIVASQRSLLEVMAAFPSAKPPLGVFFAAIAPRLQPRYYSISSSPRLAPSRVHVTSALVYGPTPTGRIHKGVCSTWMKNAVPAEKSHECSGAPIFIRASNFKLPSNPSTPIVMVGPGTGLAPFRGFLQERMALKEDGEELGSSLLFFGCRNRQMDFIYEDELNNFVDQGVISELIMAFSREGAQKEYVQHKMMEKAAQVWDLIKEEGYLYVCGDAKGMARDVHRTLHTIVQEQEGVSSSEAEAIVKKLQTEGRYLRDVW |
| tAtF5H | MTRRRRPPYPPGPRGWPIIGNMLMMDQLTHRGLANLAKKYGGLCHLRMGFLHMYAVSSPEVARQVLQVQDSVFSNRPATIAISYLTYDRADMAFAHYGPFWRQMRKVCVMKVFSRKRAESWASVRDEVDKMVRSVSCNVGKTINVGEQIFALTRNITYRAAFGSACEKGQDEFIRILQEFSKLFGAFNVADFIPYFGWIDPQGINKRLVKARNDLDGFIDDIIDEHMKKKENQNAVDDGDVVDTDMVDDLLAFYSEEAKLVSETADLQNSIKLTRDNIKAIIMDVMFGGTETVASAIEWALTELLRSPEDLKRVQQELAEVVGLDRRVEESDIEKLTYLKCTLKETLRMHPPIPLLLHETAEDTSIDGFFIPKKSRVMINAFAIGRDPTSWTDPDTFRPSRFLEPGVPDFKGSNFEFIPFGSGRRSCPGMQLGLYALDLAVAHILHCFTWKLPDGMKPSELDMNDVFGLTAPKATRLFAVPTTRLICAL |
| tAtF5H_mod1_ | MATRRRRPPYPPGPRGWPIIGNMLMMDQLTHRGLANLAKKYGGLCHLRMGFLHMYAVSSPEVARQVLQVQDSVFSNRPATIAISYLTYDRADMAFAHYGPFWRQMRKVCVMKVFSRKRAESWASVRDEVDKMVRSVSCNVGKTINVGEQIFALTRNITYRAAFGSACEKGQDEFIRILQEFSKLFGAFNVADFIPYFGWIDPQGINKRLVKARNDLDGFIDDIIDEHMKKKENQNAVDDGDVVDTDMVDDLLAFYSEEAKLVSETADLQNSIKLTRDNIKAIIMDVMFGGTETVASAIEWALTELLRSPEDLKRVQQELAEVVGLDRRVEESDIEKLTYLKCTLKETLRMHPPIPLLLHETAEDTSIDGFFIPKKSRVMINAFAIGRDPTSWTDPDTFRPSRFLEPGVPDFKGSNFEFIPFGSGRRSCPGMQLGLYALDLAVAHILHCFTWKLPDGMKPSELDMNDVFGLTAPKATRLFAVPTTRLICAL |
| tAtF5H_mod2_ | MAKKTSSTRRRRPPYPPGPRGWPIIGNMLMMDQLTHRGLANLAKKYGGLCHLRMGFLHMYAVSSPEVARQVLQVQDSVFSNRPATIAISYLTYDRADMAFAHYGPFWRQMRKVCVMKVFSRKRAESWASVRDEVDKMVRSVSCNVGKTINVGEQIFALTRNITYRAAFGSACEKGQDEFIRILQEFSKLFGAFNVADFIPYFGWIDPQGINKRLVKARNDLDGFIDDIIDEHMKKKENQNAVDDGDVVDTDMVDDLLAFYSEEAKLVSETADLQNSIKLTRDNIKAIIMDVMFGGTETVASAIEWALTELLRSPEDLKRVQQELAEVVGLDRRVEESDIEKLTYLKCTLKETLRMHPPIPLLLHETAEDTSIDGFFIPKKSRVMINAFAIGRDPTSWTDPDTFRPSRFLEPGVPDFKGSNFEFIPFGSGRRSCPGMQLGLYALDLAVAHILHCFTWKLPDGMKPSELDMNDVFGLTAPKATRLFAVPTTRLICAL |
| tAtF5H_mod3_ | MALLLAVFTRRRRPPYPPGPRGWPIIGNMLMMDQLTHRGLANLAKKYGGLCHLRMGFLHMYAVSSPEVARQVLQVQDSVFSNRPATIAISYLTYDRADMAFAHYGPFWRQMRKVCVMKVFSRKRAESWASVRDEVDKMVRSVSCNVGKTINVGEQIFALTRNITYRAAFGSACEKGQDEFIRILQEFSKLFGAFNVADFIPYFGWIDPQGINKRLVKARNDLDGFIDDIIDEHMKKKENQNAVDDGDVVDTDMVDDLLAFYSEEAKLVSETADLQNSIKLTRDNIKAIIMDVMFGGTETVASAIEWALTELLRSPEDLKRVQQELAEVVGLDRRVEESDIEKLTYLKCTLKETLRMHPPIPLLLHETAEDTSIDGFFIPKKSRVMINAFAIGRDPTSWTDPDTFRPSRFLEPGVPDFKGSNFEFIPFGSGRRSCPGMQLGLYALDLAVAHILHCFTWKLPDGMKPSELDMNDVFGLTAPKATRLFAVPTTRLICAL |
| CipA_ tAtR1_mod_ | MINDMHPSLIKDKDMMDDVMLRSCKIIAMKIMPDKVMQVMVTVLMLDGTSEEMLLKWNLLDNRGMAIYKVLMEALCGKKDVKIGTVGKVGPLGCDYINCVEISMAWKKTTADRSGELKPLMIPKSLMAKDEDDDLDLGSGKTRVSIFFGTQTGTAEGFAKALSEEIKARYEKAAVKVIDLDDYAADDDQYEEKLKKETLAFFCVATYGDGEPTDNAARFYKWFTEENERDIKLQQLAYGVFALGNRQYEHFNKIGIVLDEELCKKGAKRLIEVGLGDDDQSIEDDFNAWKESLWSELDKLLKDEDDKSVATPYTAVIPEYRVVTHDPRFTTQKSMESNVANGNTTIDIHHPCRVDVAVQKELHTHESDRSCIHLEFDISRTGITYETGDHVGVYAENHVEIVEEAGKLLGHSLDLVFSIHADKEDGSPLESAVPPPFPGPCTLGTGLARYADLLNPPRKSALVALAAYATEPSEAEKLKHLTSPDGKDEYSQWIVASQRSLLEVMAAFPSAKPPLGVFFAAIAPRLQPRYYSISSSPRLAPSRVHVTSALVYGPTPTGRIHKGVCSTWMKNAVPAEKSHECSGAPIFIRASNFKLPSNPSTPIVMVGPGTGLAPFRGFLQERMALKEDGEELGSSLLFFGCRNRQMDFIYEDELNNFVDQGVISELIMAFSREGAQKEYVQHKMMEKAAQVWDLIKEEGYLYVCGDAKGMARDVHRTLHTIVQEQEGVSSSEAEAIVKKLQTEGRYLRDVW |
| CipB_ tAtR1 _mod_ | MIIKKDILLHEDLIVDDELKVGKVEKVNIDILSPSSVIVSLNILGVVDDFHLLLVDDKDKDKIVLLYLSLLRVLHEKLDVKVKVAKSNLTKMKYIVGVEIAWKKTTADRSGELKPLMIPKSLMAKDEDDDLDLGSGKTRVSIFFGTQTGTAEGFAKALSEEIKARYEKAAVKVIDLDDYAADDDQYEEKLKKETLAFFCVATYGDGEPTDNAARFYKWFTEENERDIKLQQLAYGVFALGNRQYEHFNKIGIVLDEELCKKGAKRLIEVGLGDDDQSIEDDFNAWKESLWSELDKLLKDEDDKSVATPYTAVIPEYRVVTHDPRFTTQKSMESNVANGNTTIDIHHPCRVDVAVQKELHTHESDRSCIHLEFDISRTGITYETGDHVGVYAENHVEIVEEAGKLLGHSLDLVFSIHADKEDGSPLESAVPPPFPGPCTLGTGLARYADLLNPPRKSALVALAAYATEPSEAEKLKHLTSPDGKDEYSQWIVASQRSLLEVMAAFPSAKPPLGVFFAAIAPRLQPRYYSISSSPRLAPSRVHVTSALVYGPTPTGRIHKGVCSTWMKNAVPAEKSHECSGAPIFIRASNFKLPSNPSTPIVMVGPGTGLAPFRGFLQERMALKEDGEELGSSLLFFGCRNRQMDFIYEDELNNFVDQGVISELIMAFSREGAQKEYVQHKMMEKAAQVWDLIKEEGYLYVCGDAKGMARDVHRTLHTIVQEQEGVSSSEAEAIVKKLQTEGRYLRDVW |
| CipA_ tAtF5H | MINDMHPSLIKDKDMMDDVMLRSCKIIAMKIMPDKVMQVMVTVLMLDGTSEEMLLKWNLLDNRGMAIYKVLMEALCGKKDVKIGTVGKVGPLGCDYINCVEISMTRRRRPPYPPGPRGWPIIGNMLMMDQLTHRGLANLAKKYGGLCHLRMGFLHMYAVSSPEVARQVLQVQDSVFSNRPATIAISYLTYDRADMAFAHYGPFWRQMRKVCVMKVFSRKRAESWASVRDEVDKMVRSVSCNVGKTINVGEQIFALTRNITYRAAFGSACEKGQDEFIRILQEFSKLFGAFNVADFIPYFGWIDPQGINKRLVKARNDLDGFIDDIIDEHMKKKENQNAVDDGDVVDTDMVDDLLAFYSEEAKLVSETADLQNSIKLTRDNIKAIIMDVMFGGTETVASAIEWALTELLRSPEDLKRVQQELAEVVGLDRRVEESDIEKLTYLKCTLKETLRMHPPIPLLLHETAEDTSIDGFFIPKKSRVMINAFAIGRDPTSWTDPDTFRPSRFLEPGVPDFKGSNFEFIPFGSGRRSCPGMQLGLYALDLAVAHILHCFTWKLPDGMKPSELDMNDVFGLTAPKATRLFAVPTTRLICAL |
| CipB_ tAtF5H | MIIKKDILLHEDLIVDDELKVGKVEKVNIDILSPSSVIVSLNILGVVDDFHLLLVDDKDKDKIVLLYLSLLRVLHEKLDVKVKVAKSNLTKMKYIVGVEITRRRRPPYPPGPRGWPIIGNMLMMDQLTHRGLANLAKKYGGLCHLRMGFLHMYAVSSPEVARQVLQVQDSVFSNRPATIAISYLTYDRADMAFAHYGPFWRQMRKVCVMKVFSRKRAESWASVRDEVDKMVRSVSCNVGKTINVGEQIFALTRNITYRAAFGSACEKGQDEFIRILQEFSKLFGAFNVADFIPYFGWIDPQGINKRLVKARNDLDGFIDDIIDEHMKKKENQNAVDDGDVVDTDMVDDLLAFYSEEAKLVSETADLQNSIKLTRDNIKAIIMDVMFGGTETVASAIEWALTELLRSPEDLKRVQQELAEVVGLDRRVEESDIEKLTYLKCTLKETLRMHPPIPLLLHETAEDTSIDGFFIPKKSRVMINAFAIGRDPTSWTDPDTFRPSRFLEPGVPDFKGSNFEFIPFGSGRRSCPGMQLGLYALDLAVAHILHCFTWKLPDGMKPSELDMNDVFGLTAPKATRLFAVPTTRLICAL |

**Figure S1**. Prediction of transmembrane regions for membrane proteins (a) ATR1, (b) *At*F5H and (c) *Af*B8N.

(a) Transmembrane regions of AtR1 (Uniprot ID: Q9SB48): 27-46.

**
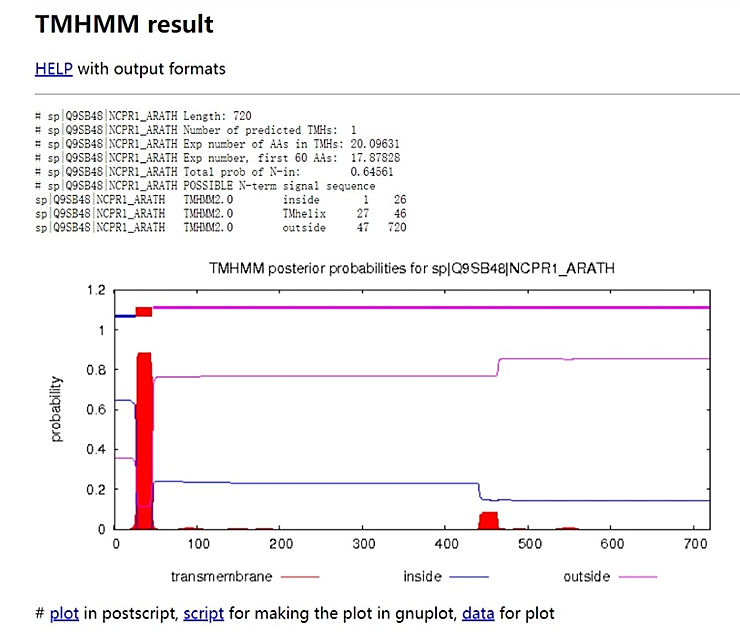
**

(b) Transmembrane regions of *At*F5H (Uniprot ID: Q9C543): 15-32.

**
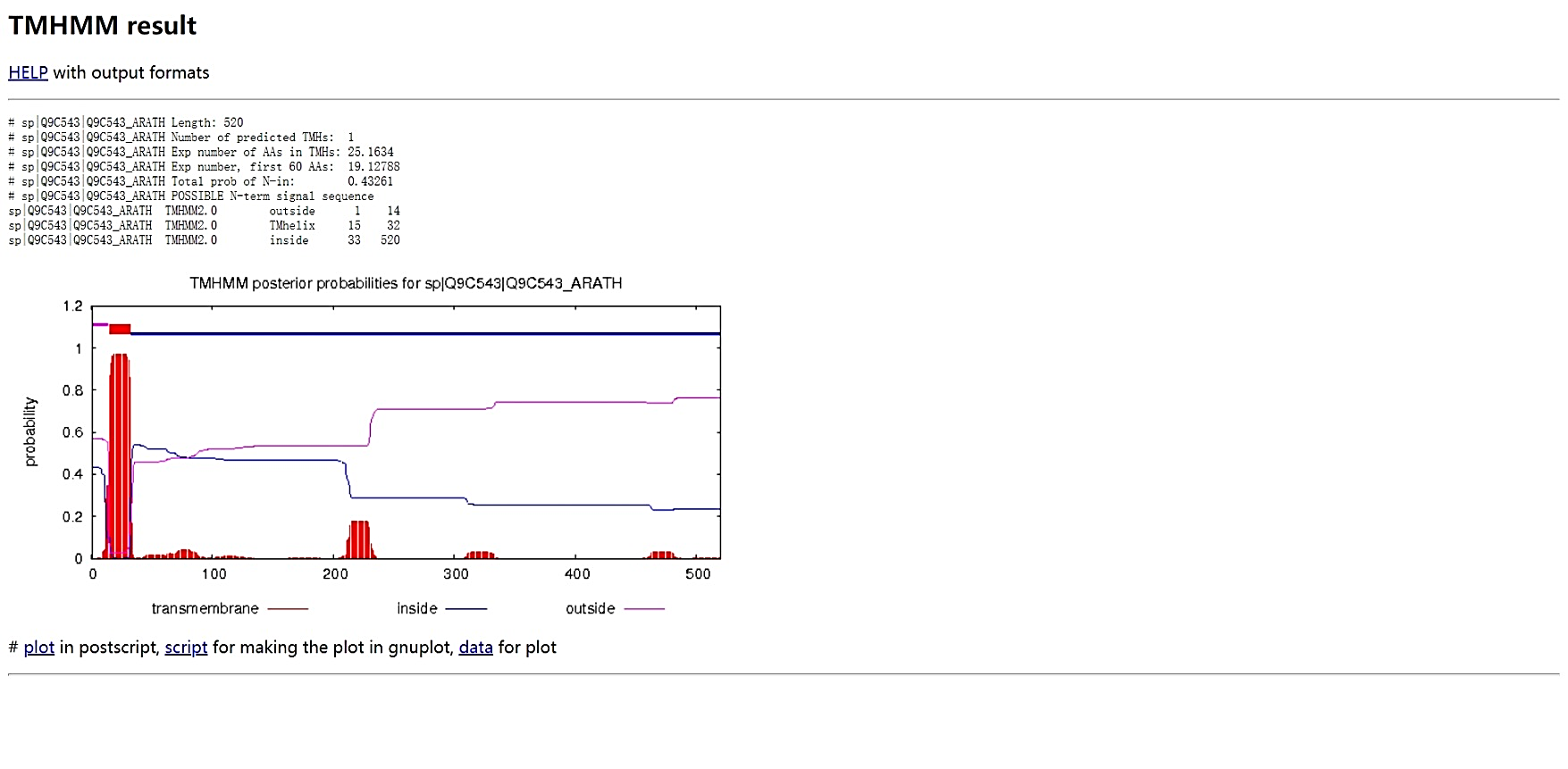
**

(c) Transmembrane regions of *Af*B8N (Uniprot ID: B8NU02): 7-29, 176-198, 205-227,232-254, and 291-313.

**
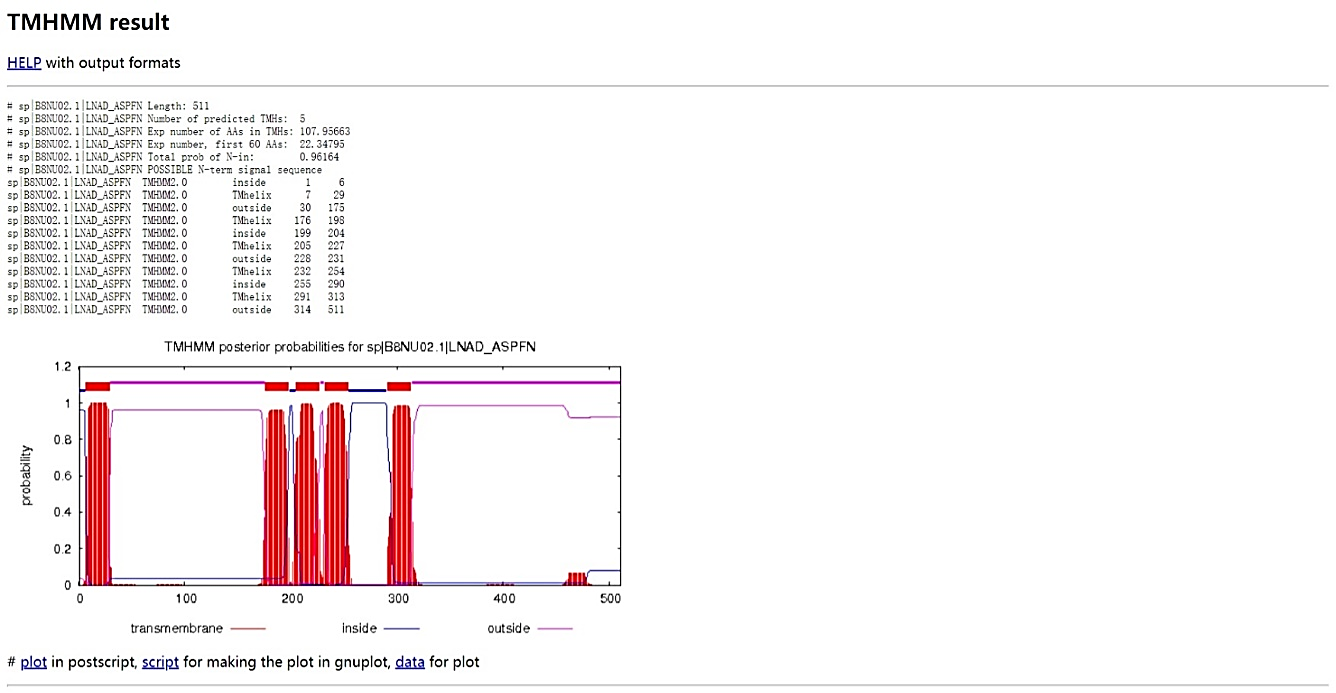
**

Figure S2. Western Blot (WB) analysis of *At*F5H_mod_s.

(Note: A 6×His-tag was fused to the C-terminus of *At*F5H_mod_s in strains tCA, 1CA, 2CA, and 3CA by Genewiz here.)


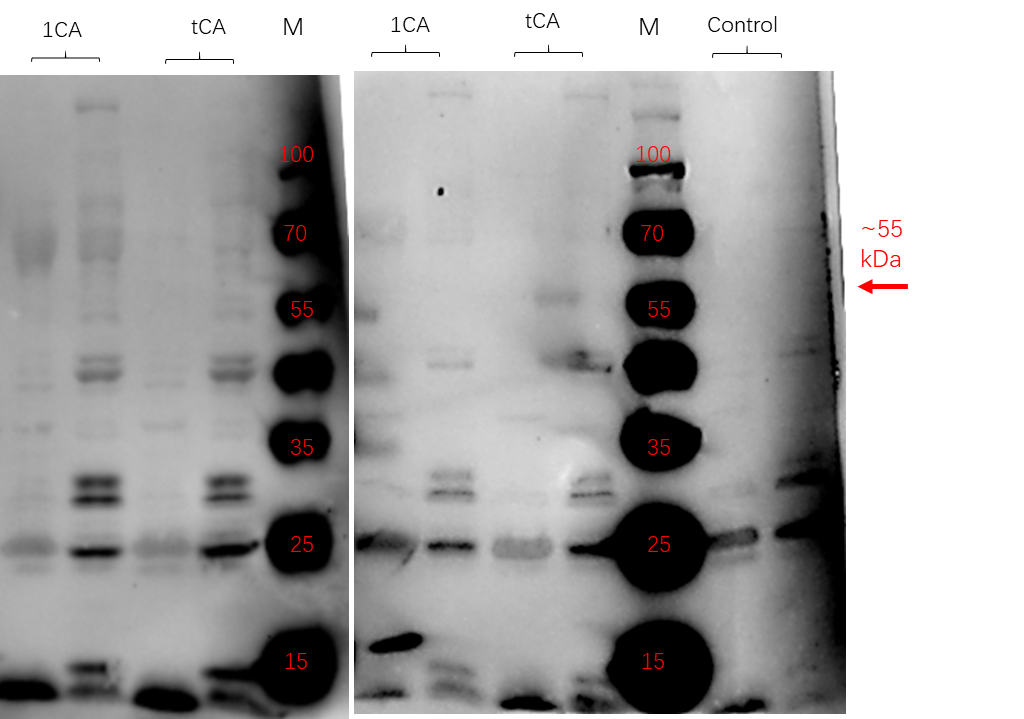


(a) WB analysis of strains Control, tCA, and 1CA.(M: Protein Marker; Left: Precipitation fractions; Right: Supernatant fractions)

**
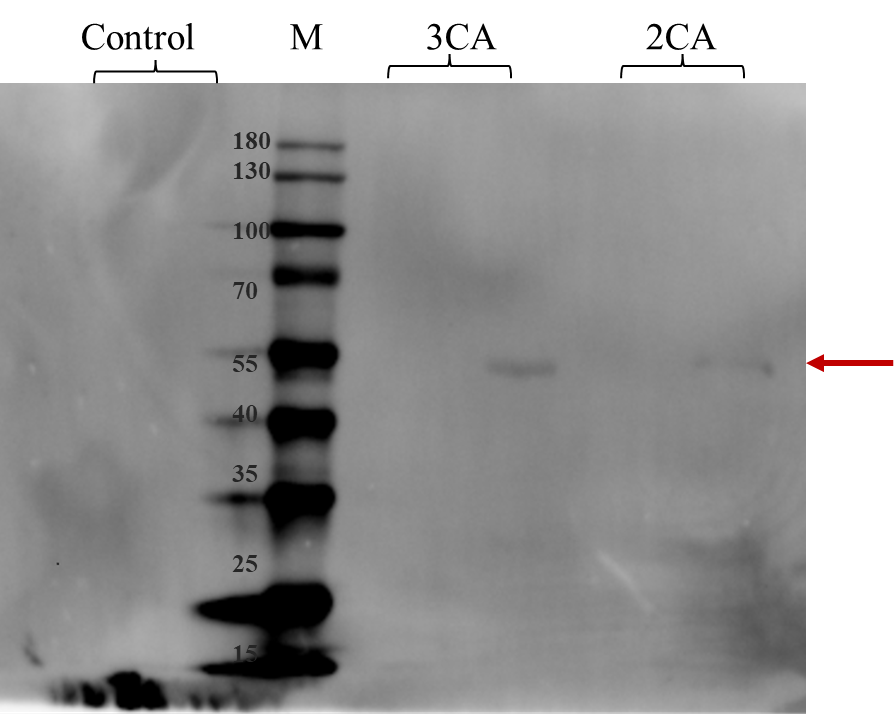
**

(b) WB analysis of strains Control, 3CA, and 2CA. (M: Protein Marker; Left: Supernatant fractions; Right: Precipitation fractions)
